# Prognostic Value and Biological Function of Galectins in Malignant Glioma

**DOI:** 10.3389/fonc.2022.834307

**Published:** 2022-06-24

**Authors:** Hongtao Zhu, Dan Liu, Lidong Cheng, Jingdian Liu, Guanghui Wang, Huan Li, Yang Zhang, Hailong Mi, Suojun Zhang, Kai Shu, Xingjiang Yu

**Affiliations:** ^1^Department of Neurosurgery, Tongji Hospital, Tongji Medical College, Huazhong University of Science and Technology, Wuhan, China; ^2^Department of Histology and Embryology, School of Basic Medicine, Tongji Medical College, Huazhong University of Science and Technology, Wuhan, China; ^3^Department of Medical Genetics, School of Basic Medicine, Tongji Medical College, Huazhong University of Science and Technology, Wuhan, China

**Keywords:** malignant glioma, galectin, LGALS, immunosuppression, M2-TAMs, PN-MES transition, stemness, hypoxia

## Abstract

Malignant glioma is the most common solid tumor of the adult brain, with high lethality and poor prognosis. Hence, identifying novel and reliable biomarkers can be advantageous for diagnosing and treating glioma. Several galectins encoded by LGALS genes have recently been reported to participate in the development and progression of various tumors; however, their detailed role in glioma progression remains unclear. Herein, we analyzed the expression and survival curves of all LGALS across 2,217 patients with glioma using The Cancer Genome Atlas (TCGA), Chinese Glioma Genome Atlas (CGGA), and Rembrandt databases. By performing multivariate Cox analysis, we built a survival model containing *LGALS1*, *LGALS3*, *LGALS3BP*, *LGALS8*, and *LGALS9* using TCGA database. The prognostic power of this panel was assessed using CGGA and Rembrandt datasets. ESTIMATE and CIBERSORT algorithms confirmed that patients in high-risk groups exhibited significant stromal and immune cell infiltration, immunosuppression, mesenchymal subtype, and isocitrate dehydrogenase 1 (IDH1) wild type. Gene Ontology (GO), Kyoto Encyclopedia of Genes and Genomes (KEGG), CancerSEA, and Gene Set Enrichment Analysis (GSEA) showed that pathways related to hypoxia, epithelial-to-mesenchymal transition (EMT), stemness, and inflammation were enriched in the high-risk group. To further elucidate the function of LGALS in glioma, we performed immunohistochemical staining of tissue microarrays (TMAs), Western blotting, and cell viability, sphere formation, and limiting dilution assays following lentiviral short hairpin RNA (shRNA)-mediated LGALS knockdown. We observed that LGALS expression was upregulated in gliomas at both protein and mRNA levels. LGALS could promote the stemness maintenance of glioma stem cells (GSCs) and positively correlate with M2-tumor-associated macrophages (TAMs) infiltration. In conclusion, we established a reliable survival model for patients with glioma based on LGALS expression and revealed the essential roles of LGALS genes in tumor growth, immunosuppression, stemness maintenance, pro-neural to mesenchymal transition, and hypoxia in glioma.

## Introduction

Glioma is the most frequently reported primary tumor of the adult central nervous system, and its treatment has been largely unsuccessful. Most patients experience inevitable relapse and finally progress to glioblastoma multiforme (GBM), the most lethal type of glioma, corresponding to the World Health Organization grade 4 glioma (1). For decades, the treatment for malignant glioma has involved surgical resection combined with postoperative radiotherapy and chemotherapy (2). However, even with maximal treatment, the median survival time of patients with GBM remains <15 months (3). The lack of specific biomarkers is an important factor that underlies treatment failure in gliomas. Hence, there is an urgent need to identify novel and reliable biomarkers of glioma development and progression.

Galectins are a family of proteins encoded by LGALS genes. Galectins contain a highly conserved carbohydrate recognition domain that can bind β-galactose residues (4). One remarkable feature of this family is the formation of ordered cell surface Gal-glycan lattices, which engage specific glycoconjugates *via* traditional ligand–receptor interactions (5). Numerous studies have reported the crucial functions of galectins in the progression of diverse tumor types, including lung cancer, melanoma, breast carcinomas, and digestive system tumors. Some researchers have focused on the function of galectins in gliomas (6–9). Galectin-1 and Galectin-8 have been shown to participate in glioma invasion (7–9), and Galectin-1 and Galectin-3 can reportedly promote immunosuppression in glioma microenvironments (6, 9, 10). However, a comprehensive description of the role of galectins in the glioma microenvironment and tumor progression is lacking.

In the present study, public glioma transcriptomic data from The Cancer Genome Atlas (TCGA), Chinese Glioma Genome Atlas (CGGA), and Repository for Molecular Brain Neoplasia Data (Rembrandt) were collected for systemic analyses. First, we analyzed the survival curve of all LGALS genes and identified five potential candidates that were negatively correlated with overall survival (OS) across the three databases. Novel nomograms for OS, progression-free survival (PFS), and disease-specific survival (DSS) with favorable predictive performance were constructed and validated based on the expression of these five LGALS using multivariate Cox analysis. We further confirmed the expression pattern of these genes in gliomas of different grades, subtypes, and isocitrate dehydrogenase (IDH)-mutated status. Additionally, we calculated stromal scores, immune scores, tumor purity, and immune cell infiltration for patients with high and low risks using the ESTIMATE and CIBERSORT algorithms. Immunohistochemical staining of patient tissue microarrays (TMAs) was performed to confirm the correlation between galectin expression and infiltration of M2 tumor-associated macrophages (TAMs). Next, the differential pathways and biological functions of high- versus low-risk patients were identified by combined analysis using CancerSEA, Gene Ontology (GO), Kyoto Encyclopedia of Genes and Genomes (KEGG), and Gene Set Enrichment Analysis (GSEA). Finally, to confirm the relationship between galectins and stemness maintenance, cell viability tests, sphere formation, limiting dilution assays, and Western blotting were performed in glioma stem cells (GSCs) after lentiviral short hairpin RNA (shRNA)-mediated LGALS knockdown. These analyses, along with immunohistochemical staining of TMAs from patients with glioma, verified the critical role of galectins in GSC stemness maintenance. **Supplementary Figure S1** presents a flowchart of the study. These results will contribute to an overall understanding of the function of galectins in glioma and highlight the potential role of these five galectins in glioma treatment strategies.

## Materials and Methods

### Datasets

TCGA datasets were downloaded from UCSC Xena (https://xenabrowser.net/), including GBM and LGG IlluminaHiSeq RNA-seq gene expression and GBMLGG phenotypes of 702 samples. CGGA datasets were downloaded from CGGA website (http://cgga.org.cn/download.jsp), including Illumina HiSeq mRNA Sequencing mRNAseq_693, Illumina HiSeq 2000 or 2500 mRNA Sequencing mRNAseq_325, and their corresponding clinical data. Rembrandt dataset was downloaded from CGGA website (http://cgga.org.cn/download_other.jsp), which included gene expression (mRNA microarray) data and clinical data of 475 glioma patients.

### Galectins Kaplan–Meier Survival Curves

Kaplan–Meier estimator survival analysis of five galectins mRNA (*LGALS*1, *LGALS*3, *LGALS*3BP, *LGALS*8, and *LGALS*9) in three datasets (TCGA, CGGA, and Rembrandt) were explored in GlioVis (gliovis.bioinfo.cnio.es/). We think one gene is correlated with patients’ survival if its log-rank p-value <0.05.

### CIBERSORT

After separating glioma patients into high- and low-risks groups based on their LGALS genes expression, we scored 22 immune cell types for their relative abundance in each glioma patient sample using CIBERSORT algorism (https://cibersort.stanford.edu/).

### ESTIMATE

By applying the R package ESTIMATE (https://bioinformatics.mdanderson.org/estimate/), we calculated the individual immune, stromal, estimate scores, and tumor purity to predict the level of immune cells and stromal cells infiltration in each glioma sample. We compared each score between patients with high and low LGALS expression.

### GSEA

Gene set enrichment analysis (GSEA) is a method to study micro-array data at the gene level. In this study, GSEA was made used in determining the differential pathways between high and low LGALS expression in glioma patients. We use the GSEA4.1.0 version to perform enrichment analysis in this study. Finally, we sorted all enriched pathways according to the nominal p-value and normalized enrichment score.

### CancerSEA

The potential role of LGALSs in glioma microenvironments was determined by CancerSEA (http://biocc.hrbmu.edu.cn/CancerSEA/) at the single-cell level. Briefly, five LGALS genes (*LGALS1*, *LGALS3*, *LGALS3BP*, *LGALS8*, and *LGALS9*) alone and together were analyzed in CancerSEA analysis tool, respectively, and their correlation with each important hallmarks of glioma was shown in the dot figure.

### Immunohistochemical Staining

Tissue microarrays (TMAs) including the normal brain and low- and high-grade gliomas (total 91 patients) were made by PiNuoFei Bio Inc. Immunohistochemical (IHC) staining of TMAs were performed using the 3,3′-diaminobenzidine (DAB) staining method. After deparaffinization and antigen retrieval, TMAs were incubated with primary antibodies at 4°C overnight. Then, DAB (Origiene, Wuxi, China) and hematoxylin (Servicebio, Wuhan, China) were used to show positive expression sites and cell nucleus, respectively. The positive stain ratio of each LGALS in glioma patients was determined by IHC Profiler, an open-source plugin for the quantitative evaluation and automated scoring of immunohistochemistry images of human tissue samples (11). Primary antibodies used for IHC included anti-Gal-1 (Proteintech, Rosemont, USA 11858-1-AP, 1:1,000), anti-Gal-3 (Proteintech, 14979-1-AP, 1:1,000), anti-Gal-3BP (Proteintech, 10281-1-AP, 1:1,000), anti-Gal-8 (Invitrogen, Carlsbad, Amercian MA5-34693, 1:100; Santa Cruz, sc-377133, 1:50), anti-Gal-9 (Proteintech, 17938-1-AP, 1:1,000), anti-Arg1 (Proteintech, 16001-1-AP, 1:100), anti-CD206 (Abcam, ab64693, 1:100), anti-Iba1 (Abcam, ab5076, 1: 200), anti-SOX2 (Abcam, ab171380, 1:200), and anti-CD15 (CST, # 4744, 1:200).

### Cell Culture and Plasmids

The primary GSC cell T387 was a kind gift from Dr. Jeremy Rich (UCSD) and Dr. Shideng Bao (Cleveland clinic) and was isolated from primary GBM cell maintained in Neurobasal A medium (Thermo Fisher, Waltham, Amercian) with B27 Supplement without vitamin A (Life Technologies, Carlsbad, Amercian), epidermal growth factor (20 ng/ml, R&D) and basic fibroblast growth factor (20 ng/ml, R&D, Minnesota, Amercian) at 37°C in 5% CO_2_. The plasmids used in this study are listed in **Supplementary Table S1**.

### Western Blotting

Briefly, cells were lysed in radioimmunoprecipitation assay (RIPA) lysis buffer supplemented with protease and phosphatase inhibitor cocktails (MCEs). After protein quantitation of each sample using the bicinchoninic acid (BCA) assay, protein samples were resolved by sodium dodecyl sulfate–polyacrylamide gel electrophoresis (SDS-PAGE) and transferred onto polyvinylidene fluoride (PVDF) membranes. Membranes were then incubated with primary antibodies overnight at 4°C followed by horseradish peroxidase (HRP)-conjugated rabbit/mouse-specific antibodies (Jackson ImmunoResearch West Grove, USA, 1:10,000). All immunoblot results were independently repeated at least three times. Primary antibodies used for immunoblot were listed as follows: anti-Gal-1 (Proteintech, 11858-1-AP, 1:1,000), anti-Gal-3 (Proteintech, 14979-1-AP, 1:1,000), anti-Gal-3BP (Proteintech, 10281-1-AP, 1:1,000), anti-Gal-8 (Invitrogen, MA5-34693, 1:500), anti-Gal-9 (Proteintech, 17938-1-AP, 1:10,00), anti-cleaved poly(ADP-ribose) polymerase (PARP) (CST, #5625S, 1:1,000), anti-cleaved caspase3 (CST, #9664S, 1:1,000); anti-Olig2 (Proteintech, 66513-1-Ig, 1:1,000); anti-SOX2 (Abcam, Cambridge, England ab171380, 1:1,000); anti-α-tubulin (ABclonal, Wuhan, China AC012, 1:5,000).

### Cell Viability Assay

Cell viability assay was conducted using CellTiter-Lum Plus Cell Viability Assay kit (Beyotime, Shanghai, China) according to the manufacturer’s guidance. Briefly, 1 × 10^3^ cells of each group were plated into each well of 96-well plates. Cell titers were determined at the 0, 1, 3, and 5 days after seeding cells. All data were performed in triplicate and normalized to day 0 and presented as mean ± SEM.

### Sphere Formation Assay and *In Vitro* Limit Dilution Assay

For the sphere formation assay, GSCs were implanted into 96-well plates at a density of 1 × 10^3^ cells per well. After incubation for 5 days, tumorsphere numbers were calculated. For *in vitro* limiting dilution assay, GSCs were plated in a 96-well plate at a gradient of 1, 10, 20, 30, 40, and 50 cells per well, with eight replicates in each group. Seven days post-implantation, the tumorspheres in each well was determined under a microscope; the Extreme Limiting Dilution Analysis tool (http://bioinf.wehi.edu.au/software/elda) was used to calculate the sphere formation efficiency of GSCs in each group.

### Lentivirus Package and Lentiviral shRNA-Based Gene Knockdown

shRNA hairpins targeting human *LGALS1*, *LGALS3*, *LGALS3BP*, *LGALS8*, and *LGALS9* were used in this study. For each gene, we screened at least three shRNAs in the pLKO.1 vector and select two of them that reduced protein levels by >80%. For these selected shRNAs, we performed lentivirus package in 293T cells. Briefly, 7.5 mg of the psPAX2 plasmid, 7 mg of the pMD2.G plasmid, and 8 mg of the shRNA plasmid were transfected into 293T cells in 100-mm dishes using calcium phosphate precipitation. The supernatants containing lentivirus were collected 72 h post-transfection and filtered through a 0.45-μm filter (Biosharp, Hefei, China). Cells were infected with viral supernatant containing 8 mg/ml polybrene (Sigma, Darmstadt, Germany) and then selected using 1 mg/ml puromycin (Thermo Fisher). The knockdown efficiency of each shRNA was validated by Western blotting.

### Statistics

GraphPad Prism 8 software was used for statistical analysis charting. All results were presented as the mean ± SEM unless otherwise stated. p < 0.05 was considered statistically significant. R software version 3.6.3 (R Core Team, R Foundation for Statistical Computing, Vienna, Austria) was used for the rest statistical analyses. R package limma was used to determine differential genes (DEGs) of micro-array data; R package DEseq2 was used to gain DEGs of RNA-seq data. For gene-set enrichment analysis and visualization, we use ClusterProfiler package (12).

## Results

### LGALS Expression Correlates With Survival in Patients With Glioma

**Figure 1:**
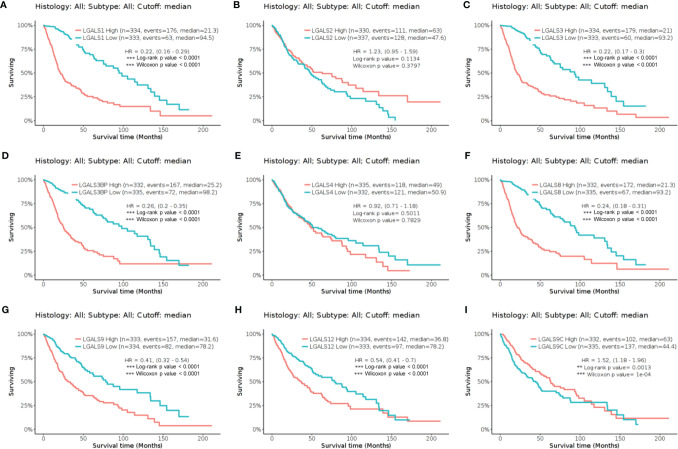
The Kaplan–Meier curve of LGALS genes in TCGA Glioma dataset. **(A)** The Kaplan–Meier curve of *LGALS1* in TCGA Glioma dataset. **(B)** The Kaplan–Meier curve of *LGALS2* in TCGA Glioma dataset. **(C)** The Kaplan–Meier curve of *LGALS3* in TCGA Glioma dataset. **(D)** The Kaplan–Meier curve of *LGALS3BP* in TCGA Glioma dataset. **(E)** The Kaplan–Meier curve of *LGALS4* in TCGA Glioma dataset. **(F)** The Kaplan–Meier curve of *LGALS8* in TCGA Glioma dataset. **(G)** The Kaplan–Meier curve of *LGALS9* in TCGA Glioma dataset. **(H)** The Kaplan–Meier curve of *LGALS12* in TCGA Glioma dataset. **(I)** The Kaplan–Meier curve of *LGALS9C* in TCGA Glioma dataset. TCGA, The Cancer Genome Atlas **p<0.01, ***p<0.0001.

Sixteen LGALS genes are known to encode the galectin family protein. To determine the roles of galectins in glioma, we first analyzed the relationship between LGALS mRNA expression and survival in patients with glioma. Using GlioVis website tools (gliovis.bioinfo.cnio.es/) (13), we found that *LGALS1*, *LGALS3*, *LGALS3BP*, *LGALS8*, *LGALS9*, *LGALS9C*, and *LGALS12* expression levels negatively correlated with patient survival in TCGA dataset (**Figures 1A–I**). We further validated these results in the CGGA (**Supplementary Figure S2**) and Rembrandt datasets (**Supplementary Figure S3**) and found that the expression of *LGALS1*, *LGALS3*, *LGALS3BP*, *LGALS8*, and *LGALS9* was significantly correlated with patient survival in all three datasets.

### Survival Model Containing Five LGALS Genes Affords Reliable Prognostic Power to Predict Survival in Patients With Glioma

**Figure 2:**
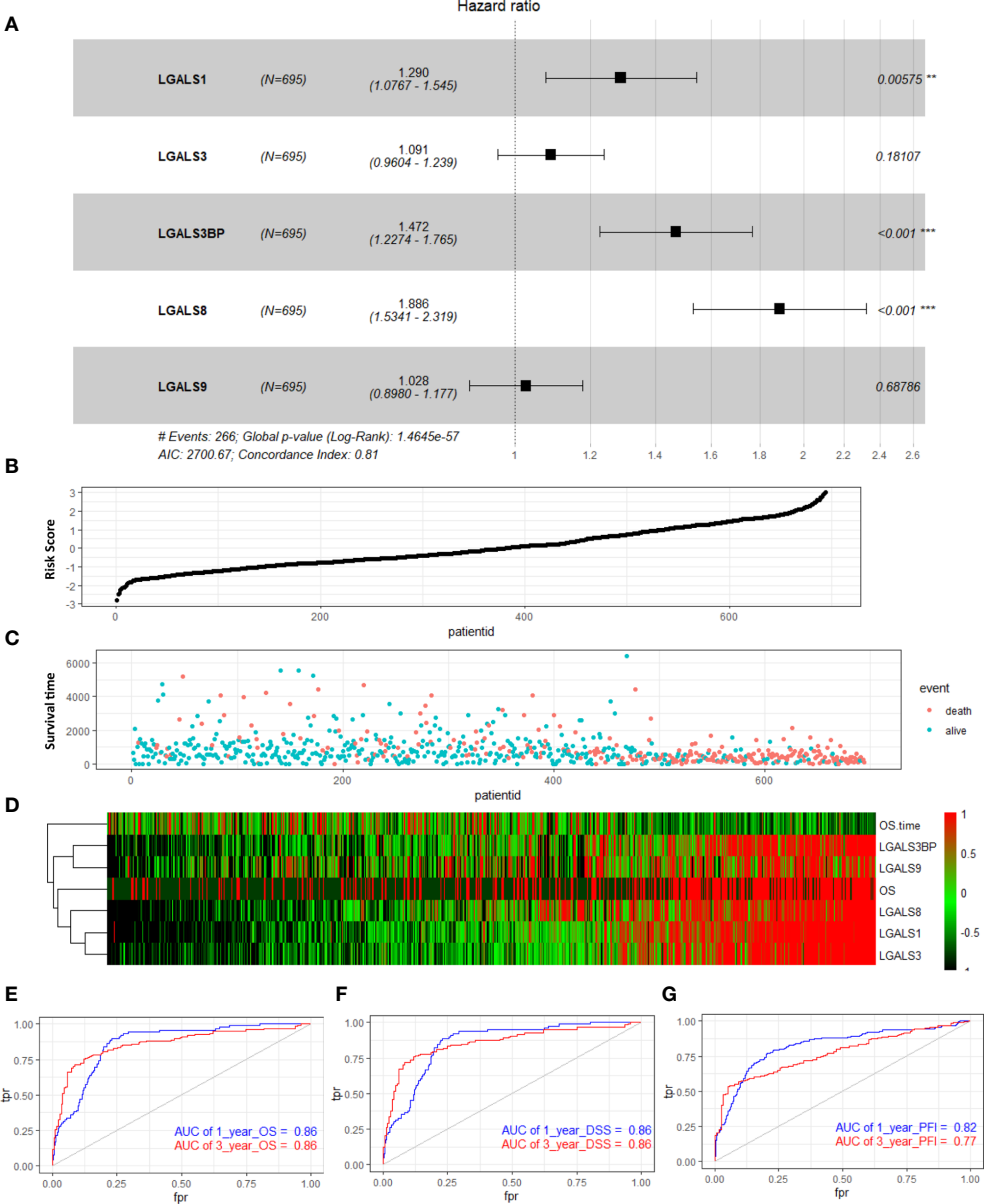
Distribution of risk score, survival status, and risk heatmap of LGALS in patients with glioma revealed by multivariable Cox regression analysis of TCGA Glioma dataset. **(A)** Multivariable Cox regression analysis based on the five LGALSs expressions. The black and solid squares represent the HR of death. Close-ended horizontal lines represent 95% CI. **(B)** The risk score curve of the LGALSs signature. **(C)** Patient survival status and time distributed by risk score. **(D)** Heatmaps of the expression levels of the 5 LGALSs and OS time of glioma patients. The colors from green to red indicate the expression level from low to high. **(E–G)** The prognostic performance of the LGALSs signature demonstrated by the time-dependent ROC curve for predicting the 1- and 3-year OS **(E)**, DSS **(F),** and PFI **(G)** rates in the TCGA Glioma dataset. TCGA, The Cancer Genome Atlas **p<0.01, ***p<0.001.

To further investigate the prognostic value of these LGALS genes in glioma, we performed multivariate Cox analyses on a new risk signature comprising these five genes in all patients of the TCGA GBMLGG RNA-seq dataset. The risk score was calculated for each patient in the TCGA GBMLGG cohort, and all patients were divided into high-risk (high-risk score) and low-risk (low-risk score) groups using the median value of the risk score as the cutoff. The C-index of this signature was 0.81 (log-rank p = 1.4654e−57, **Figure 2A**). With an increase in the patient risk score, the expression of these five genes increased, and the OS gradually decreased (**Figures 2B–D**). According to time-dependent receiver operating characteristic curve analysis results, the LGALS signature showed excellent values in predicting 1- and 3-year OS rates in the TCGA GBMLGG dataset, with respective area under curve (AUC) values of 0.86 (**Figure 2E**). Moreover, the AUCs for the 1- and 3-year DSS and PFS rates using the prognostic model were 0.86 and 0.86 (DSS, **Figure 2F**), and 0.82 and 0.77 (PFS, **Figure 2G**), respectively. A similar multivariate Cox regression was performed using the CGGA and Rembrandt glioma cohorts, and positive results were acquired. The C-index of this signature in CGGA and Rembrandt was 0.65 (log-rank p = 5.6521e−30, **Supplementary Figure S4A**) and 0.68 (log-rank p = 1.1347e−18, **Supplementary Figure S5A**), respectively. The AUCs for the 1- and 3-year OS were 0.68 and 0.71 (**Supplementary Figure S4E**) in CGGA and 0.69 and 0.84 in Rembrandt (**Supplementary Figure S5E**), respectively. These results indicated that the LGALS gene signature might serve as a reliable prognostic model for survival in patients with glioma.

### Galectins Are Highly Expressed in Glioma Tissue, and LGALS Expression Risk Scores Correlate With Glioma Grades, Subtypes, and IDH Mutation Status

**Figure 3:**
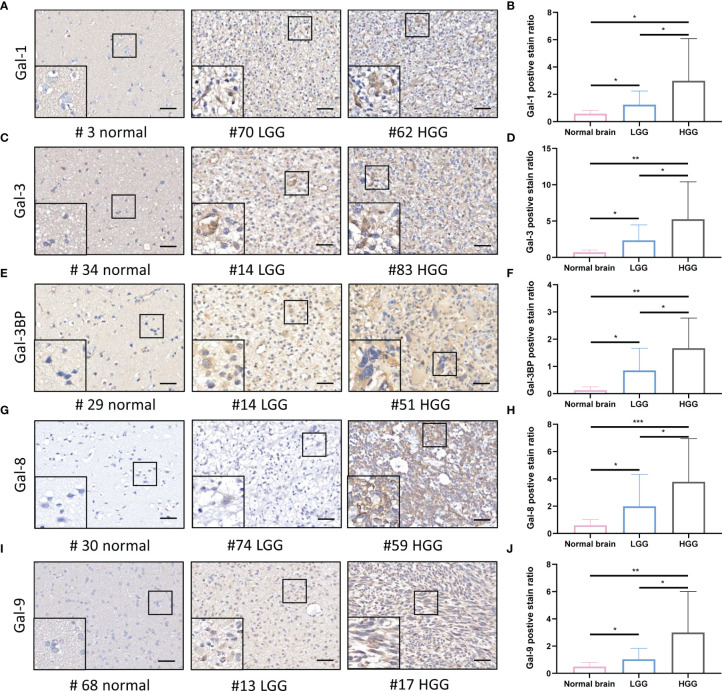
Galectin expression levels in the human normal brain and LGG and HGG tissues as shown by IHC staining. **(A)** Immunohistochemical staining of Galectin1 (Gal-1) in glioma patients TMA. Representative staining images of the normal brain; LGG and HGG are shown. **(B)** The positive stain ratio of Gal-1 in TMA glioma specimens, the statistical chart of **Figure 3A**. Similarly, the representative IHC staining images of Gal-3 **(C)** and statistical chart of its positive stain ratio **(D)**, the representative IHC staining images of Gal-3BP **(E)** and statistical chart of its positive stain ratio **(F)**, the representative IHC staining images of Gal-8 **(G)** and statistical chart of its positive stain ratio **(H)**, the representative IHC staining images of Gal-9 **(I)**, and statistical chart of its positive stain ratio **(J)** were shown. The scale bar measures 40 μm. IHC, immunohistochemistry; HGG, high-grade glioma; LGG, low-grade glioma *p<0.05, **p<0.01, ***p<0.001.

**Figure 4:**
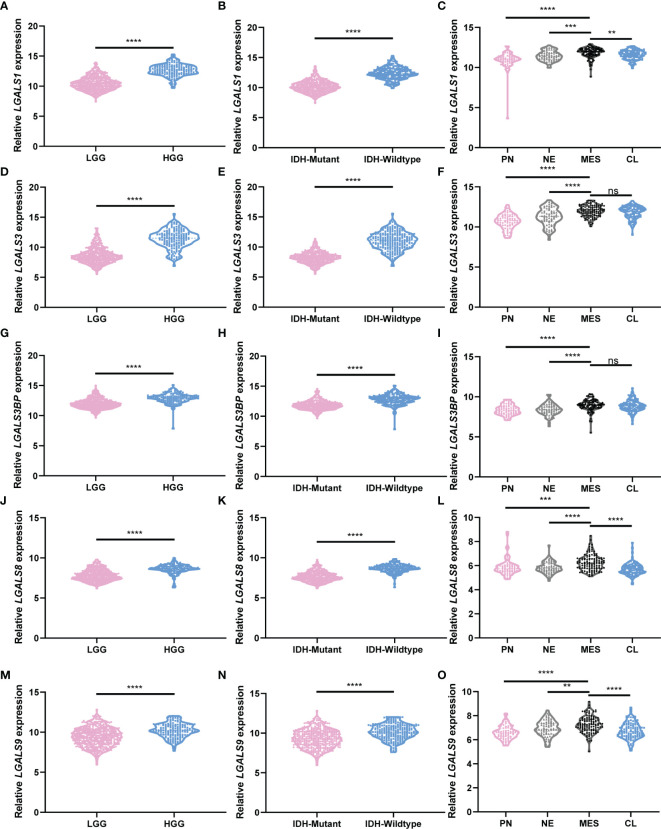
LGALS expression correlates with glioma grades, GBM subtypes, and IDH mutation status in TCGA Glioma dataset. **(A)** Relative *LGALS*1 expression level of LGG and HGG patients in TCGA Glioma dataset. **(B)** Relative *LGALS*1 expression level of IDH-mutant and IDH-wild-type patients in TCGA Glioma dataset. **(C)** Relative *LGALS*1 expression level of different molecular subtype patients in TCGA Glioma dataset. **(D–F)** Relative *LGALS*3 expression level of LGG and HGG patients **(D)**, IDH-mutant and IDH-wild-type patients **(E)**, and different molecular subtype patients **(F)** in TCGA Glioma dataset. **(G–I)** Relative *LGALS*3BP expression level of LGG and HGG patients **(G)**, IDH-mutant and IDH-wild-type patients **(H)**, and different molecular subtype patients **(I)** in TCGA Glioma dataset. **(J–L)** Relative *LGALS*8 expression level of LGG and HGG patients **(J)**, IDH-mutant and IDH-wild-type patients **(K)**, and different molecular subtype patients **(L)** in TCGA Glioma dataset. **(M–O)** Relative *LGALS*9 expression level of LGG and HGG patients **(M)**, IDH-mutant and IDH-wild-type patients **(N)**, and different molecular subtype patients **(O)** in TCGA Glioma dataset. IDH, isocitrate dehydrogenase; GBM, glioblastoma; TCGA, The Cancer Genome Atlas **p<0.01, ***p<0.001, ****p<0.0001, ns, No significance.

To comprehensively clarify the galectin expression patterns in glioma, we performed immunohistochemical (IHC) staining of TMAs for each galectin (**Figures 3A, C, E, G, I****)**. An open-source plugin for the quantitative evaluation of immunohistochemistry images, IHC Profiler, was employed to calculate the positive stain ratio of each LGALS in the TMAs of patients with glioma. We observed that protein levels of all five galectins were upregulated in gliomas when compared with normal brain tissue and positively correlated with patient grades (**Figures 3B, D, H, F, J****)**. The Human Protein Atlas (HPA) database was used to further validate the expression features of the five galectins. IHC staining in the HPA database revealed that five galectins were significantly upregulated in glioma tissues when compared with the normal brain cortex, and the protein level gradually increased with tumor grade (**Supplementary Figure S6A–J**). Next, we separated patients with glioma into several subgroups based on their IDH mutation status and subtypes (Verhaak (14) for CGGA and Rembrandt datasets and Phillips (15) for TCGA cohort). We noted that the mesenchymal subtype, representing the most aggressive type with the highest invasion and worst survival, exhibited higher LGALS expression than other subtypes (**Figures 4C, F, I, L, O**; **Supplementary Figures S7C, F, I, L, O**, **S8B, D, F, H, J**). Moreover, patients with wild-type IDH exhibited higher levels of LGALS mRNA expression than IDH mutations (**Figures 4B, E, H, K, N****;** **Supplementary Figures S7B, E, H, K, N**). LGALS mRNA expression in patients with glioma also increased with patient grades in each examined database (**Figures 4A, D, G, J, M**; **Supplementary Figures S7A, D, G, J, M**, **S8A, C, E, G, I**). These results suggested that galectin expression might correlate with aggressive glioma phenotypes.

### LGALSs Expression Indicates Stromal Cells Infiltration and Immunosuppression in Glioma Patients

**Figure 5:**
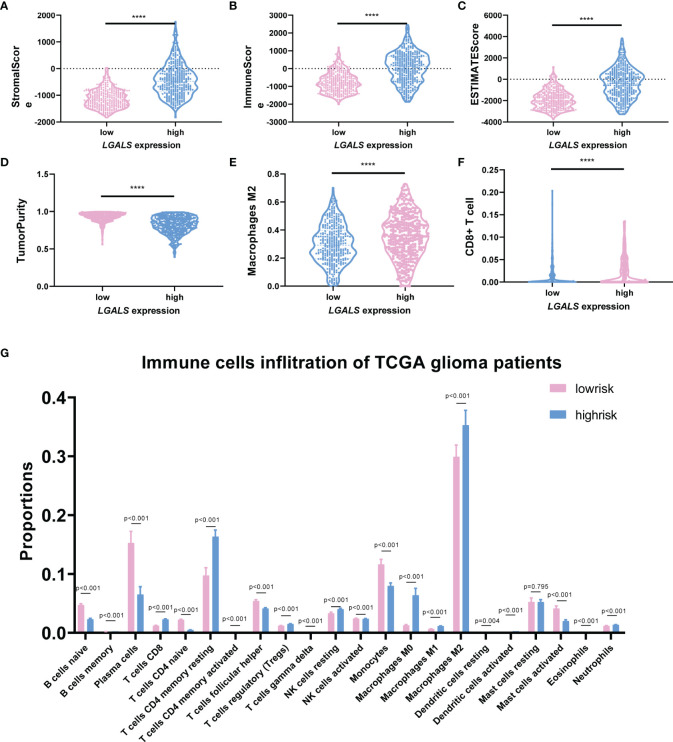
LGALS expression indicates stromal cell infiltration and immunosuppression in TCGA Glioma dataset. **(A)** The StromalScore, **(B)** ImmuneScore, **(C)** ESTIMATEScore, and **(D)** TumorPurity of glioma patients with high and low LGALS expression based on LGALS signature. **(G)** The immune cells infiltration of glioma patients with high and low LGALS expression based on LGALS signature; the proportions of 22 immune cells were calculated. The proportion of macrophage M2 **(E)** and CD8^+^ T cell **(F)** was shown separately ****p<0.0001.

**Figure 6:**
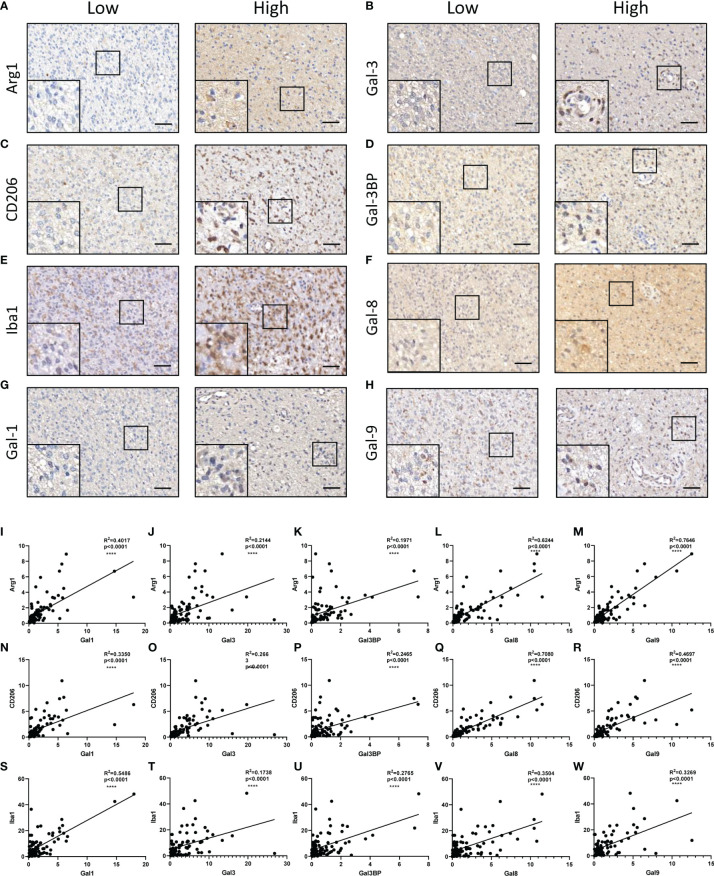
Correlations between galectins and M2-TAMs infiltration at the protein level in glioma tissues. Arg1 **(A)**, Gal-3 **(B)**, CD206 (Marker for M2-TAMs) **(C)**, Gal-3BP **(D)**, Iba1 (marker for pan TAMs) **(E)**, Gal-8 **(F)**, Gal-1 **(G)**, and Gal-9 **(H)** are shown. The scale bar measures 40 μm. **(I–M)** Pearson correlation analysis of the positive stain ratio of Arg1 and Galectins. **(N–R)** Pearson correlation analysis of the positive stain ratio of CD206 and Galectins. **(S–W)** Pearson correlation analysis of the positive stain ratio of Iba1 and Galectins. TAMs, tumor-associated macrophages ****p<0.0001.

It has been reported that Galectin-3 may contribute to immunosuppression in glioma (6, 10). We next clarified whether the constructed LGALS model could demonstrate infiltration of immune and other stromal cells in glioma microenvironments. ESTIMATE and CIBERSORT algorithms were applied to calculate stromal scores, immune scores, estimate scores, tumor purity, and infiltration of different types of immune cells for each patient in the three datasets. Data revealed that patients with high-risk scores had higher stromal scores (**Figure 5A** and **Supplementary Figures S9A**, **S10A**) and immune scores (**Figure 5B** and **Supplementary Figures S9B**, **S10B**) and lower estimate scores (**Figure 5C** and **Supplementary Figures S9C**, **S10C**) and tumor purity (**Figure 5D** and **Figures S9D**, **S10D**) than patients with low-risk scores, as it is well-known that high stromal scores, immune scores, and low tumor purity indicate worse prognosis and persistent resistance to treatments (16). In addition, among the 22 immune cells analyzed by CIBERSORT (**Figure 5G** and **Supplementary Figures S9G**, **S10G**), CD8^+^ T cell infiltration was decreased, while M2 macrophages were increased across the three datasets (**Figures 5E, F** and **Supplementary Figures S9E, F**, **S10E, F**), further demonstrating the potential role of LGALS genes in glioma immunosuppression and immune escape. Given that M2-TAMs are the most abundant immune cells in the glioma microenvironment and play essential roles in glioma progression, we performed IHC staining of markers for M2-TAMs using TMAs of patients with glioma (**Figures 6A–H**). We found that galectin expression was positively correlated with Iba1 (pan TAMs marker) and Arg1 and CD206 (M2-TAMs marker) at the protein level, as determined by Pearson correlation analysis (**Figures 6I–W**). These results indicated a strong correlation between galectin expression and M2-TAM infiltration.

### LGALSs Gene Expression Correlates With Epithelial-to-Mesenchymal Transition, Hypoxia, and Inflammation at Both Single-Cell and RNA-seq Levels

**Figure 7:**
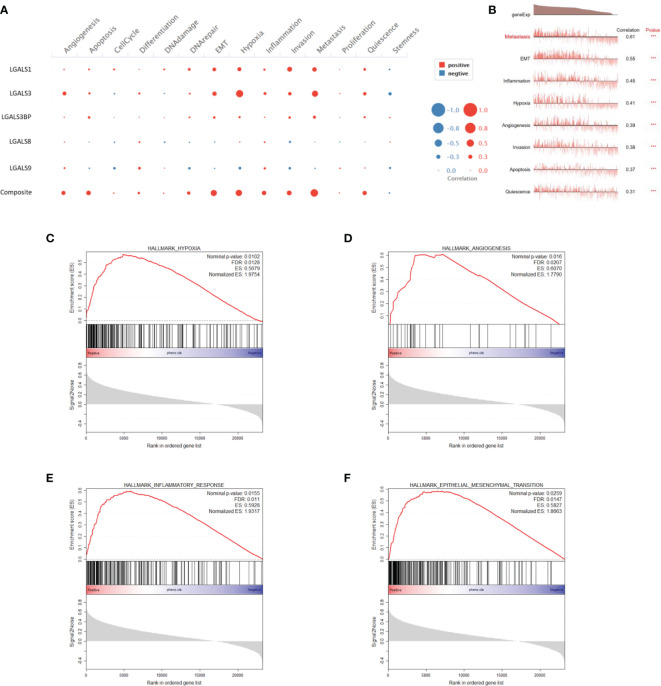
LGALS expression correlates with EMT, hypoxia, inflammation, and angiogenesis of glioma in both single-cell and RNA-seq levels. **(A)** Correlations between each LGALS expression and several biological processes in single-cell RNA-seq level. **(B)** Correlations between LGALS signature and biological processes in single-cell RNA-seq level. **(C–F)** GSEA analysis of CGGA dataset shows that pathways containing hypoxia **(C)**, angiogenesis **(D)**, epithelial-to-mesenchymal transition (EMT) **(F)**, and inflammatory response **(E)** were activated in patients with high-risk scores. EMT, epithelial-to-mesenchymal transition.

The refractory nature of glioma is largely attributed to its heterogeneity, which has been greatly uncovered by single-cell RNA-seq in recent years (17). Next, we used CancerSEA, a database that reveals the distinct functional states of cancer cells at a single-cell resolution (18), to explore the role of LGALS genes at the single-cell level. We found that LGALS expression correlated with epithelial-to-mesenchymal transition (EMT), hypoxia, inflammation, angiogenesis, invasion, apoptosis, and quiescence in gliomas (**Figures 7A, B**). GSEA was also performed using CGGA and TCGA RNA-seq data for further validation. As expected, GSEA showed that pathways involving hypoxia, angiogenesis, EMT, and inflammatory response were activated in patients with high-risk scores (high LGALSs expression) (**Figures 7C–F**).

### Galectins Promote GSC Stemness Maintenance and Proliferation *In Vitro*

**Figure 8:**
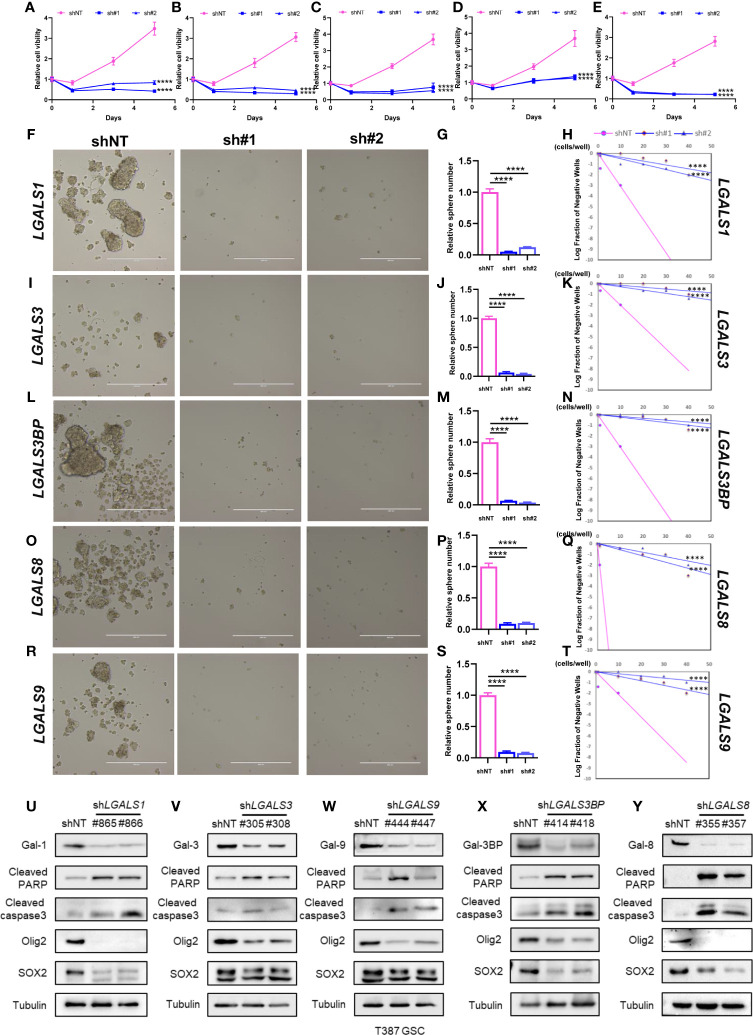
Galectins promote GSC stemness maintenance and proliferation *in vitro*. **(A–E)** The cell viability assay of T387 GSC under *LGALS1* **(A)**, *LGALS3* **(B)**, *LGALS3BP* **(C)**, *LGALS8* **(D)**, and *LGALS9* **(E)** knockdown. **(F–T)** Sphere formation assay and *in vitro* limiting dilution assay of GSC under LGALSs knockdown. T387 GSC was transduced with two different shRNA sequences against *LGALS1* **(F)**, *LGALS3* **(I)**, *LGALS3BP* **(L)**, *LGALS8* **(O)**, and *LGALS9* **(R)** and incubated for 5 days, then tumorspheres were assessed by bright-field microscopy. Relative quantification of tumorspheres in T387 GSC after *LGALS*s knockdown is shown in panels **(G, J, M, P, S)**. *In vitro* limiting dilution assays were performed in T387 GSC expressed shNT and sh*LGALS1* **(H)**, sh*LGALS3* **(K)**, sh*LGALS3BP* **(N)**, sh*LGALS8* **(Q)**, and sh*LGALS9* **(T)**. **(U–Y)** Western blotting analysis of cleaved PARP, cleaved caspase3, Oligo2, and SOX2 proteins in T387 GSC with *LGALS1* **(U)**, *LGALS3* **(V)**, *LGALS3BP* **(W)**, *LGALS8* **(X)**, and *LGALS9* **(Y)** knockdown. GSC, glioma stem cell ****p<0.0001.

**Figure 9:**
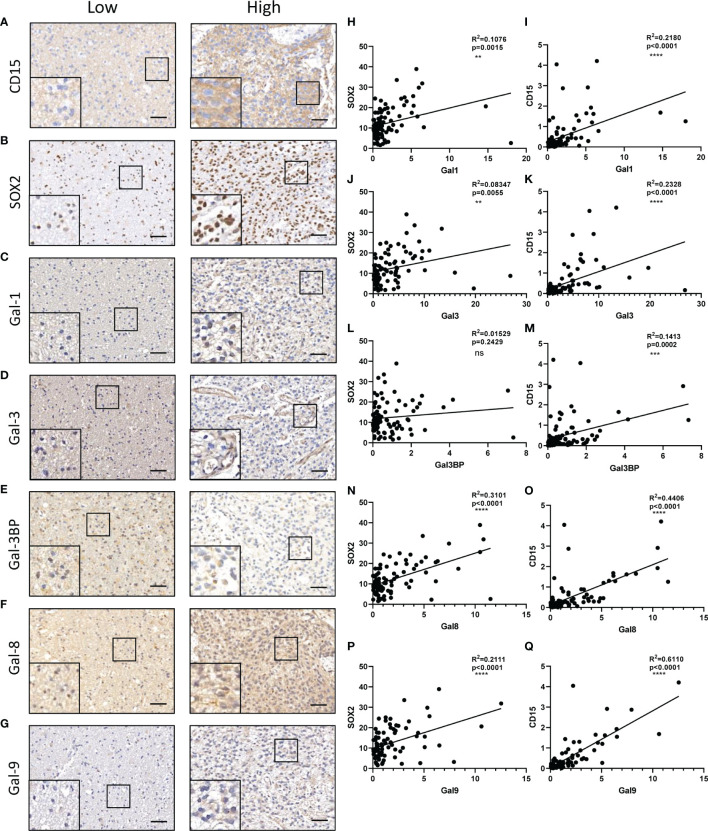
Correlations between galectins and GSC content at the protein level in glioma tissues. CD15 **(A)**, SOX2 (marker for GSCs) **(B)**, Gal-1 **(C)**, Gal-3 **(D)**, Gal-3BP **(E)**, Gal-8 **(F)**, and Gal-9 **(G)** are shown. The scale bar measures 40 μm. **(H, J, L, N, P)** Pearson correlation analysis of the positive stain ratio of SOX2 and Galectins. **(I, K, M, O, Q)** Pearson correlation analysis of the positive stain ratio of CD15 and Galectins. GSC, glioma stem cell **p<0.01, ***p<0.001, ****p<0.0001, ns, No significance.

It has been reported that galectin expression is highly correlated with tumor stemness in lung cancer, ovarian cancer, renal cell carcinoma, hepatocellular carcinomas, and melanoma (19–23); however, the function of galectins in glioma stemness remains elusive. Herein, we performed lentiviral shRNA knockdown to investigate the potential role of LGALS in GSC maintenance. Cell viability assays showed that tumor growth was significantly impaired upon downregulation of these five LGALS genes (**Figures 8A–E**). Tumor sphere formation assays and *in vitro* limiting dilution assays indicated that LGALS genes are crucial for the self-renewal capacity of GSC (**Figures 8F–T**). To further confirm the essential role of LGALS in GSC stemness maintenance and proliferation, Western blotting was performed to determine protein levels of molecular markers of cell apoptosis and stemness. Higher expression levels of cleaved PARP and cleaved caspase3 and lower SOX2 and Oligo2 expression were observed in GSCs transfected with shRNA against LGALS than in the control group (shNT) (**Figures 8U–Y**). Collectively, these data demonstrated that LGALS genes are crucial for GSC maintenance and proliferation *in vitro*. To further investigate the roles of galectins (proteins coded by LGALS genes) in glioma stemness maintenance in patient tumor specimens, we performed IHC staining of GSC markers using patient TMAs (**Figures 9A–G**). As expected, galectin expression was positively correlated with CD15 and SOX2 (markers for GSCs) at the protein level, as determined by Pearson correlation analysis (**Figures 9H–Q**).

### Glioma Patients With High-Risk Scores Exhibit Enhanced Activation of Several Important Signaling Pathways

**Figure 10:**
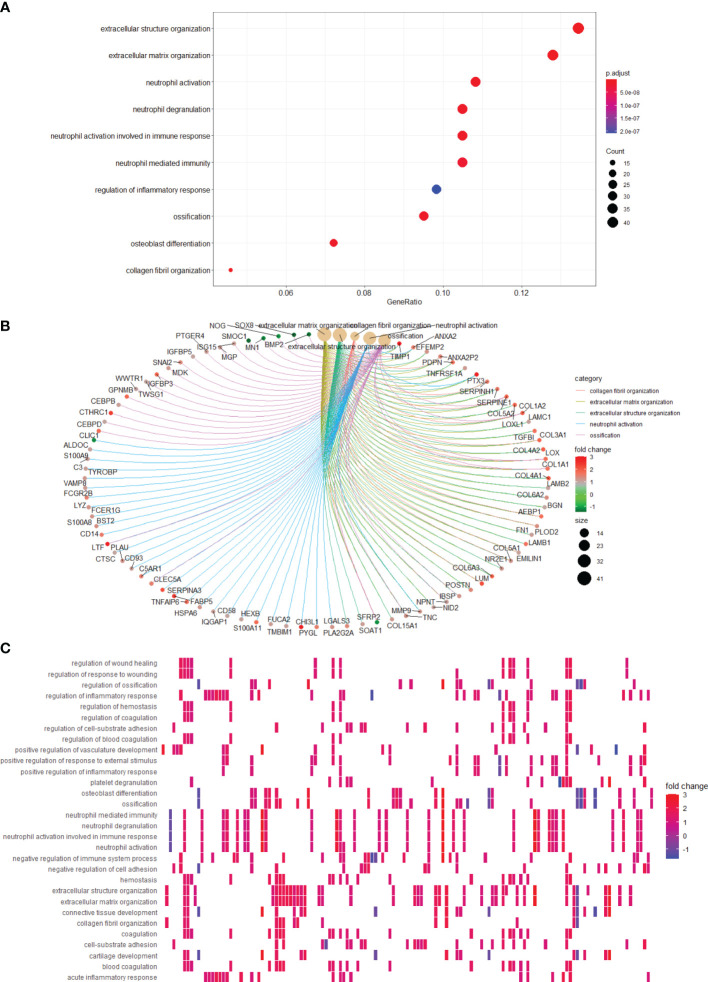
Pathways correlating with LGALS expression. **(A)** Pathway enrichment analysis showed high LGALSs expression correlated with several pathways. **(B)** Genes that belong to top 5 pathways are shown. **(C)** Pathways correlate with high LGALS expression.

To gain insight into the functional role of LGALS genes in glioma, we utilized GO and KEGG databases to define the differential pathways correlated with LGALS gene expression and used the ClusterProfiler R package for visualization. High LGALS expression correlated with activation of extracellular structure organization, extracellular matrix organization, and regulation of the inflammatory response (**Figure 10A**). Genes that belong to each pathway are shown in **Figure 10B**. Genes common to the top 5 activated pathways include POSTN, MMP9, CEBPB, CEBPD, and IGFBP5 (**Figure 10C**), which reportedly participate in glioma growth, invasion, and immunosuppression (24–28).

## Discussion

Treatment of malignant gliomas remains unsuccessful (29, 30). Currently, postoperative strategies are mainly non-specific, including temozolomide (TMZ) and radiotherapy (31). Drugs that specifically target glioma cells and induce fewer side effects on normal cells are lacking. The highly refractory nature of this tumor can be attributed to its high intra- and inter-tumoral heterogeneities, which hinder the establishment of distinctive targets that are appropriate for most patients with glioma (17). Furthermore, relevant cell lines or samples from patients with glioma included in a traditional single study fail to sufficiently neutralize the intra- and inter-glioma heterogeneities. Multi-omics sequencing and downstream bioinformatics analysis can provide a solution, as numerous patients can be analyzed to identify signatures with better universality.

Galectin, encoded by LGALS genes, can reportedly participate in the progression of various tumors, but its role in glioma remains poorly understood. Herein, we used a series of *in silico* methods to determine the function of galectins in gliomas. Using IHC results of TMAs from patients with glioma and RNA-seq data from the TCGA project, we found five upregulated galectins in glioma tissues when compared with normal brain tissue, and their expression negatively correlated with patient survival time. Moreover, multivariable Cox regression demonstrated that the five LGALS genes exhibited excellent prognostic and predictive values for OS, DSS, and PFS. These results were validated in CGGA and Rembrandt patient cohorts.

Previous studies have shown that immunosuppression is a prominent hallmark of gliomas, which are mainly composed of M2 subtype macrophages (32). In the present study, we confirmed that patients with high LGALS expression had higher stromal scores, immune scores, and M2 macrophage levels and lower estimate scores, tumor purity, and CD8^+^ T cells than those with low expression, as determined using the ESTIMATE and CIBERSORT algorithms. The correlations between galectin expression and M2-TAM infiltration were further validated by IHC staining of TMAs of patients with glioma. However, the detailed molecular mechanisms need to be comprehensively investigated.

Verhaak and Phillips classified malignant gliomas into several molecular subtypes based on their transcriptome expression patterns (14, 15). Among them, the pro-neural subtype has lower invasiveness and portends a more favorable prognosis, whereas the mesenchymal subtype exhibits overt aggressiveness and worst OS. Elucidating the core molecules and pathways of the pro-neural to mesenchymal (PN-MES) transition is crucial for developing novel therapeutic strategies. Herein, we found that all five LGALS genes were highly expressed in the mesenchymal subtype, which suggests a potential role of galectins in glioma PN-MES transition.

GSCs represent a subpopulation of glioma cells exhibiting unique surface markers and play key roles in resistance to chemotherapy and radiotherapy in patients with glioma (33, 34). It has been reported that galectin expression promotes tumor stemness in several cancers, but its role in glioma is still unclear. In the present study, we performed several molecular biology methods, including lentiviral shRNA knockdown, cell viability assay, sphere formation assay, *in vitro* limiting dilution assay, Western blotting, and IHC staining, to unravel the potential role of galectins in GSC stemness maintenance. We found that galectins promoted GSC stemness maintenance and proliferation *in vitro*. However, the detailed mechanisms and signaling pathways through which galectins promote GSC stemness require further investigation.

Hypoxia is another distinct feature of malignant glioma, known to induce active angiogenesis (35), which is also found to be elevated in LGALS high-expressing glioma by CancerSEA and GSEA analysis. However, owing to the extensive inter- and intra-tumoral heterogeneities (36), some contradictions can be noted in results obtained from different databases, warranting intensive investigations of each LGALS in terms of glioma progression.

Overall, we established a relatively comprehensive description of galectin expression patterns and biological functions in malignant glioma microenvironments by performing a series of bioinformatics analyses and several molecular biology methods. We believe that targeting galectins could be a novel and effective treatment strategy for malignant glioma.

## Data Availability Statement

The datasets presented in this study can be found in online repositories. The names of the repository/repositories and accession number(s) can be found in the article/**Supplementary Material**.

## Ethics Statement

The studies involving human participants were reviewed and approved by the Ethics Committee of Tongji Hospital of Tongji Medical College, Huazhong University of Science and Technology. The patients/participants provided their written informed consent to participate in this study.

## Author Contributions

HZ, DL, SZ, and LC contributed to the conception of the study. HZ, KS, and XY design this study. JL and GW organized the database. HZ, HL, YZ, and HM performed molecular biology assays. HZ wrote the manuscript and then revised by XY and KS. All authors contributed to the article and approved the submitted version.

## Funding

This work was supported by the National Natural Science Foundation of China (82072805, 81974452, and 81402058), the National Natural Science Foundation of Hubei (2020CFB657), the start-up funding of Huazhong University of Science and Technology (2019 kfyXJJS187), and Program for HUST Academic Frontier Youth Team.

## Conflict of Interest

The authors declare that the research was conducted in the absence of any commercial or financial relationships that could be construed as a potential conflict of interest.

## Publisher’s Note

All claims expressed in this article are solely those of the authors and do not necessarily represent those of their affiliated organizations, or those of the publisher, the editors and the reviewers. Any product that may be evaluated in this article, or claim that may be made by its manufacturer, is not guaranteed or endorsed by the publisher.
