# Supplementary material for: Prognostic Value and Biological Function of Galectins in Malignant Glioma
Source: Front Oncol. 2022 Jun 24;12:834307. doi: 10.3389/fonc.2022.834307 (PMC9263596; doi:10.3389/fonc.2022.834307)
Supplement: Supplementary file 1 [file DataSheet_1.pdf]

Supplementary Material

1 Supplementary Figures and Tables

1.1 Supplementary Tables

Table S1 plasmids used in this study

NAME	SOURCE	IDENTIFER
psPAX2	Addgene	Plasmid #12260
pMD2.G	Addgene	Plasmid #12259
shRNA pLKO.1 CTRL shRNA	Sigma-Aldrich	Cat# SHC002
shRNA pLKO.1 LGALS1 shRNA#1	Sigma-Aldrich	TRCN0000011865
shRNA pLKO.1 LGALS1 shRNA#2	Sigma-Aldrich	TRCN0000011866
shRNA pLKO.1 LGALS3 shRNA#1	Sigma-Aldrich	TRCN0000029305
shRNA pLKO.1 LGALS3 shRNA#2	Sigma-Aldrich	TRCN0000029308
shRNA pLKO.1 LGALS3BP shRNA#1	Sigma-Aldrich	TRCN0000029414
shRNA pLKO.1 LGALS3BP shRNA#2	Sigma-Aldrich	TRCN0000029418
shRNA pLKO.1 LGALS8 shRNA#1	Sigma-Aldrich	TRCN0000057355
shRNA pLKO.1 LGALS8 shRNA#2	Sigma-Aldrich	TRCN0000057357
shRNA pLKO.1 LGALS9 shRNA#1	Sigma-Aldrich	TRCN0000057444
shRNA pLKO.1 LGALS9 shRNA#2	Sigma-Aldrich	TRCN0000057447

1.2 Supplementary Figures

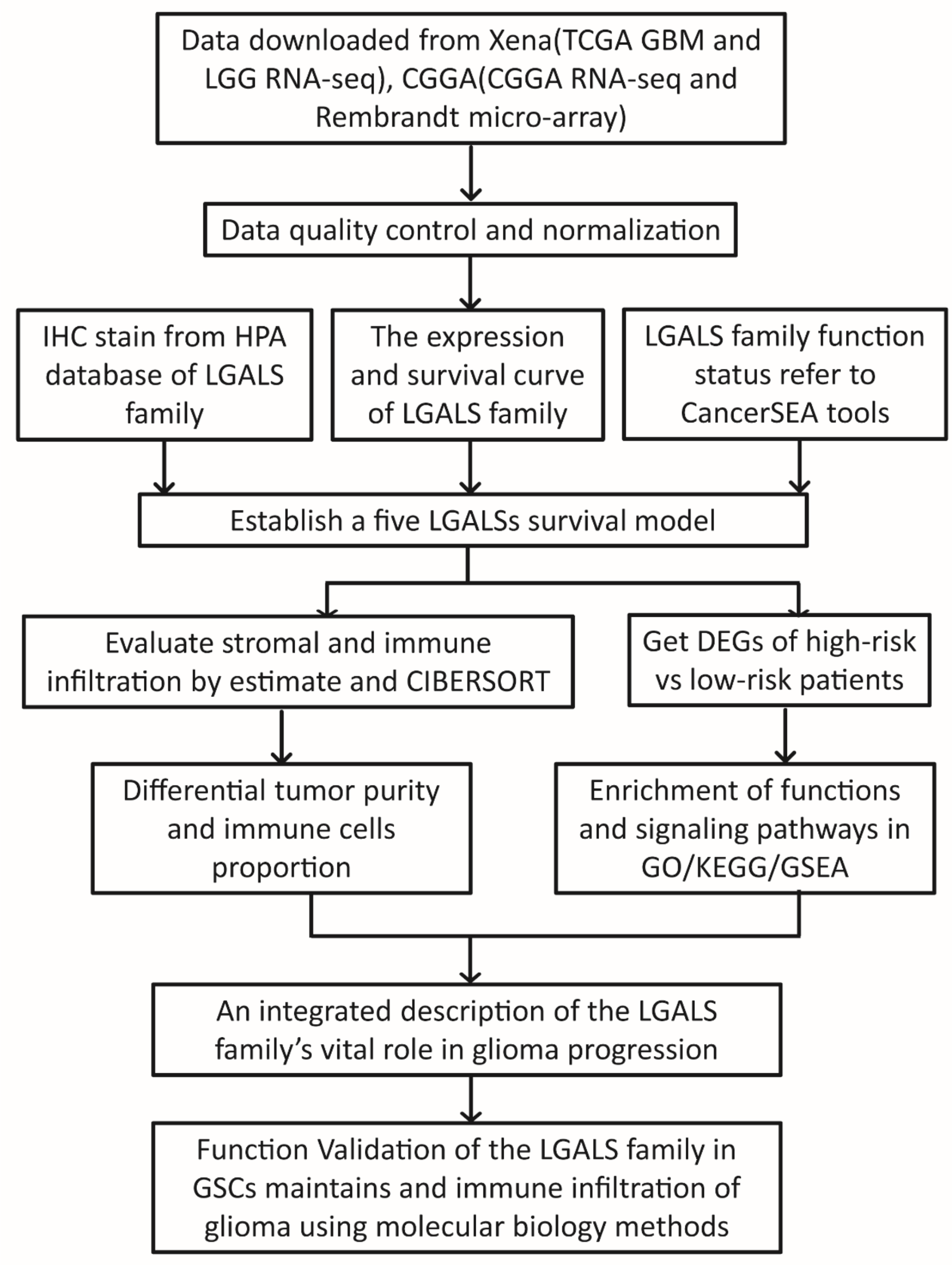

Supplementary Figure 1. Flowchart of this study.

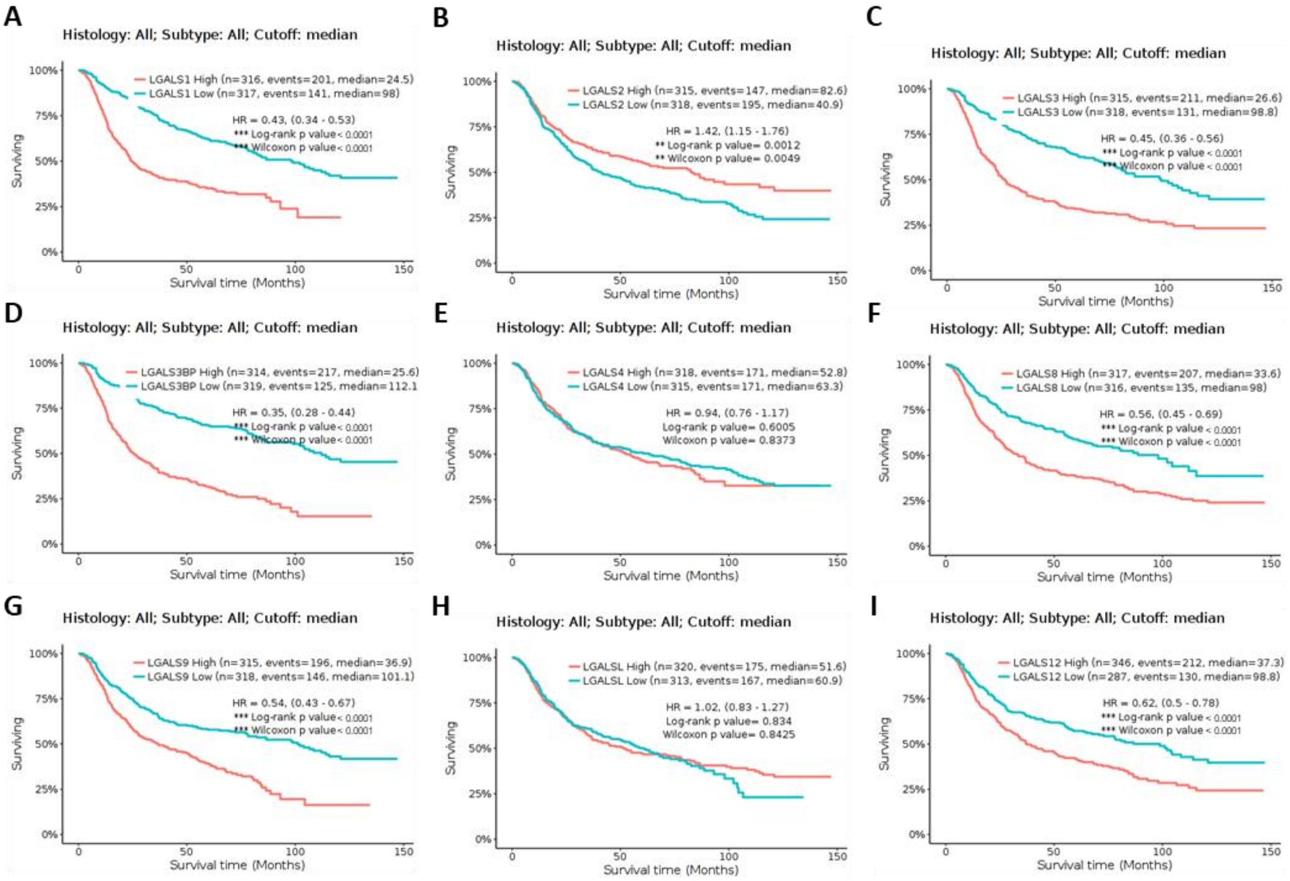

Supplementary Figure 2. The Kaplan–Meier curve of LGALS genes in CGGA Glioma dataset. **(A)** The Kaplan–Meier curve of *LGALS1* in TCGA Glioma dataset. **(B)** The Kaplan–Meier curve of *LGALS2* in TCGA Glioma dataset. **(C)** The Kaplan–Meier curve of *LGALS3* in TCGA Glioma dataset. **(D)** The Kaplan–Meier curve of *LGALS3BP* in TCGA Glioma dataset. **(E)** The Kaplan–Meier curve of *LGALS4* in TCGA Glioma dataset. **(F)** The Kaplan–Meier curve of *LGALS8* in TCGA Glioma dataset. **(G)** The Kaplan–Meier curve of *LGALS9* in TCGA Glioma dataset. **(H)** The Kaplan–Meier curve of *LGALSL* in TCGA Glioma dataset. **(I)** The Kaplan–Meier curve of *LGALS12* in TCGA Glioma dataset.

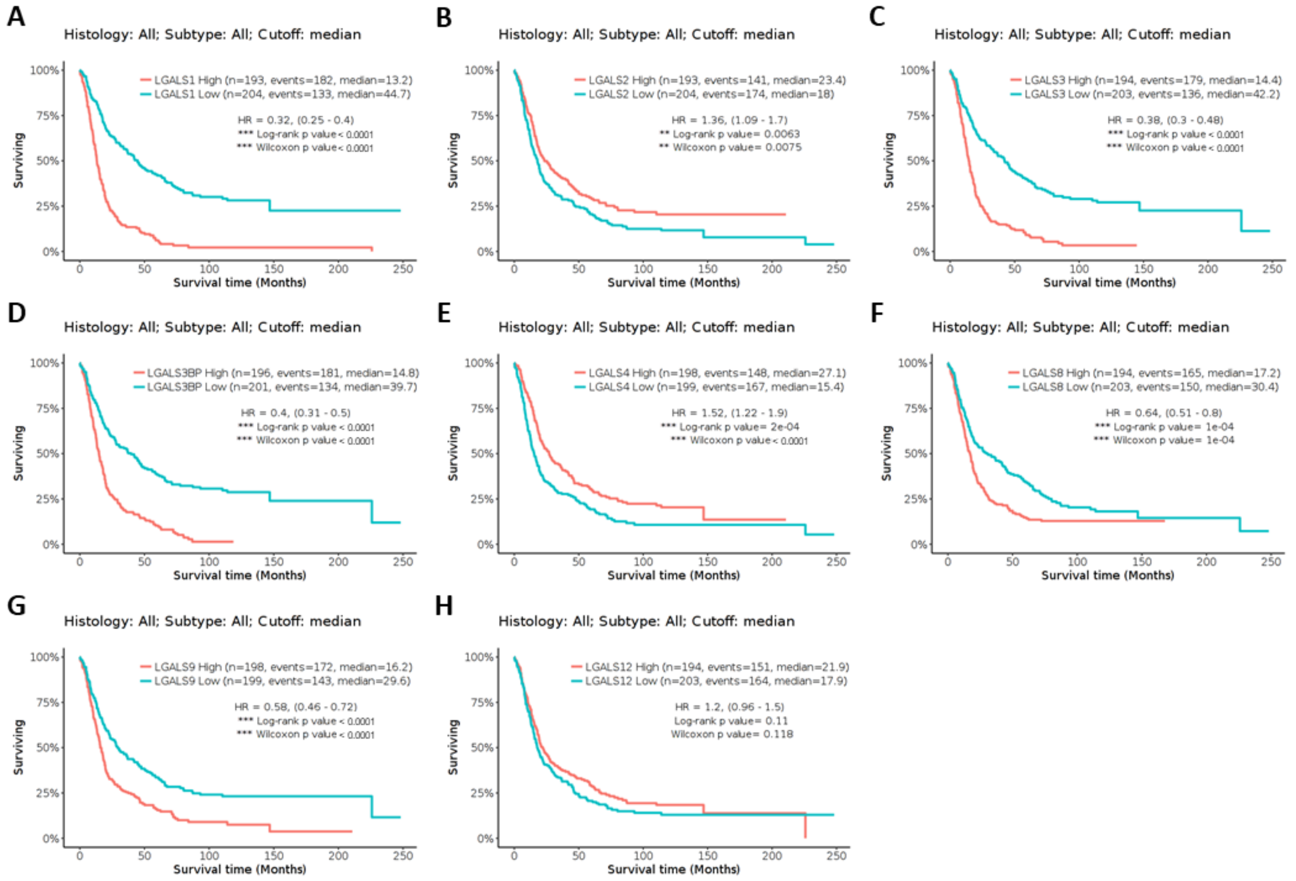

Supplementary Figure 3. The Kaplan–Meier curve of LGALS genes in Rembrandt Glioma dataset. **(A)** The Kaplan–Meier curve of *LGALS1* in TCGA Glioma dataset. **(B)** The Kaplan–Meier curve of *LGALS2* in TCGA Glioma dataset. **(C)** The Kaplan–Meier curve of *LGALS3* in TCGA Glioma dataset. **(D)** The Kaplan–Meier curve of *LGALS3BP* in TCGA Glioma dataset. **(E)** The Kaplan–Meier curve of *LGALS4* in TCGA Glioma dataset. **(F)** The Kaplan–Meier curve of *LGALS8* in TCGA Glioma dataset. **(G)** The Kaplan–Meier curve of *LGALS9* in TCGA Glioma dataset. **(H)** The Kaplan–Meier curve of *LGALS12* in TCGA Glioma dataset.

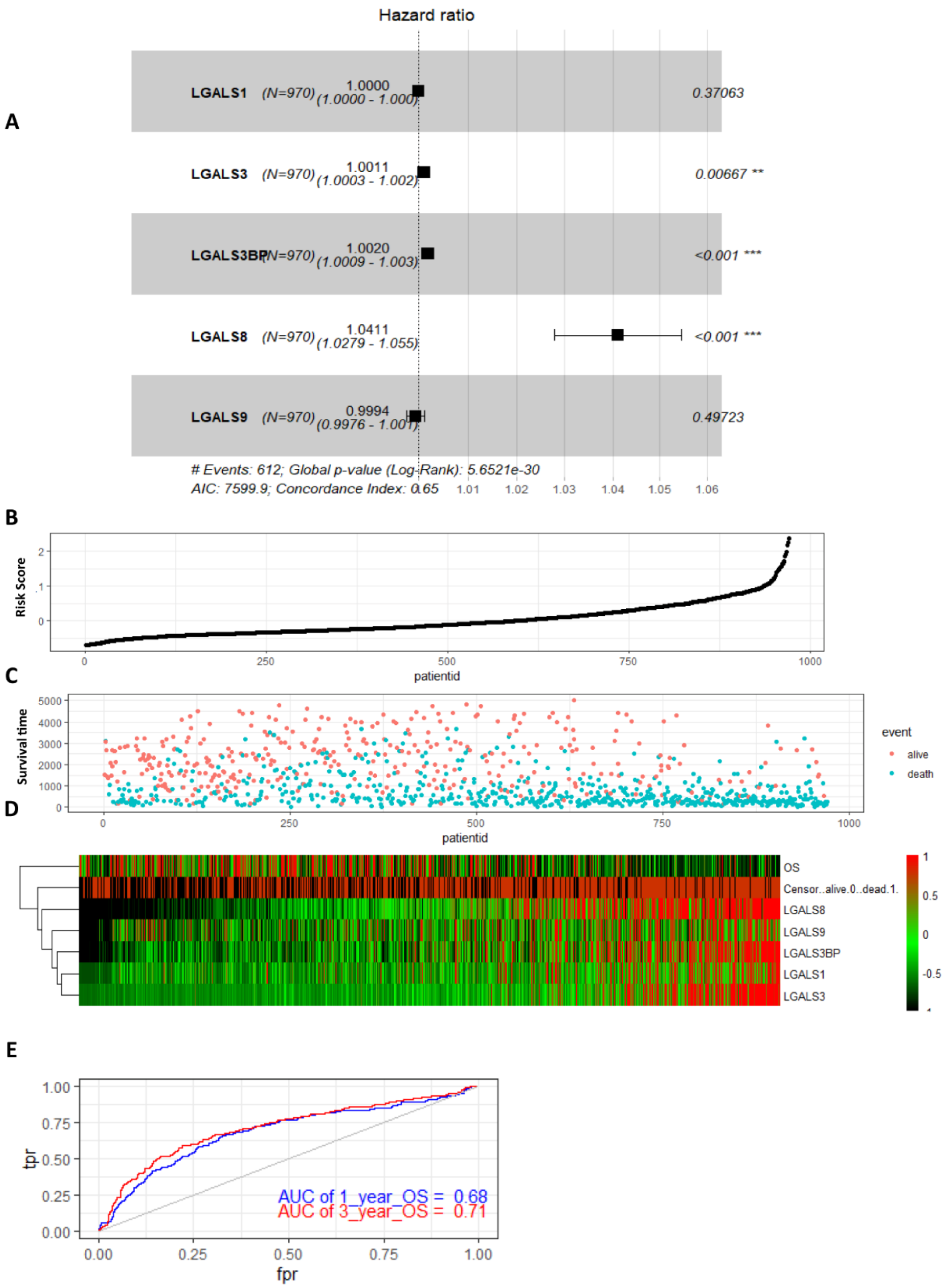

Supplementary Figure 4. Distribution of risk score, survival status, and risk heatmap of LGALS in patients with glioma revealed by multivariable Cox regression analysis of CGGA Glioma dataset. **(A)** Multivariable Cox regression analysis based on the 5 *LGALS*s expression. The black and solid squares represent the HR of death. Close-ended horizontal lines represent 95% CI. **(B)** The risk score curve of the *LGALS*s signature. **(C)** Patient survival status and time distributed by risk score. **(D)** Heatmaps of the expression levels of the 5 *LGALS*s and OS time of glioma patients. The colors from green to red indicate the expression level from low to high. **(E)** The prognostic performance of the *LGALS*s signature demonstrated by the time-dependent ROC curve for predicting the 1-, 3-year OS rates in the CGGA Glioma dataset.

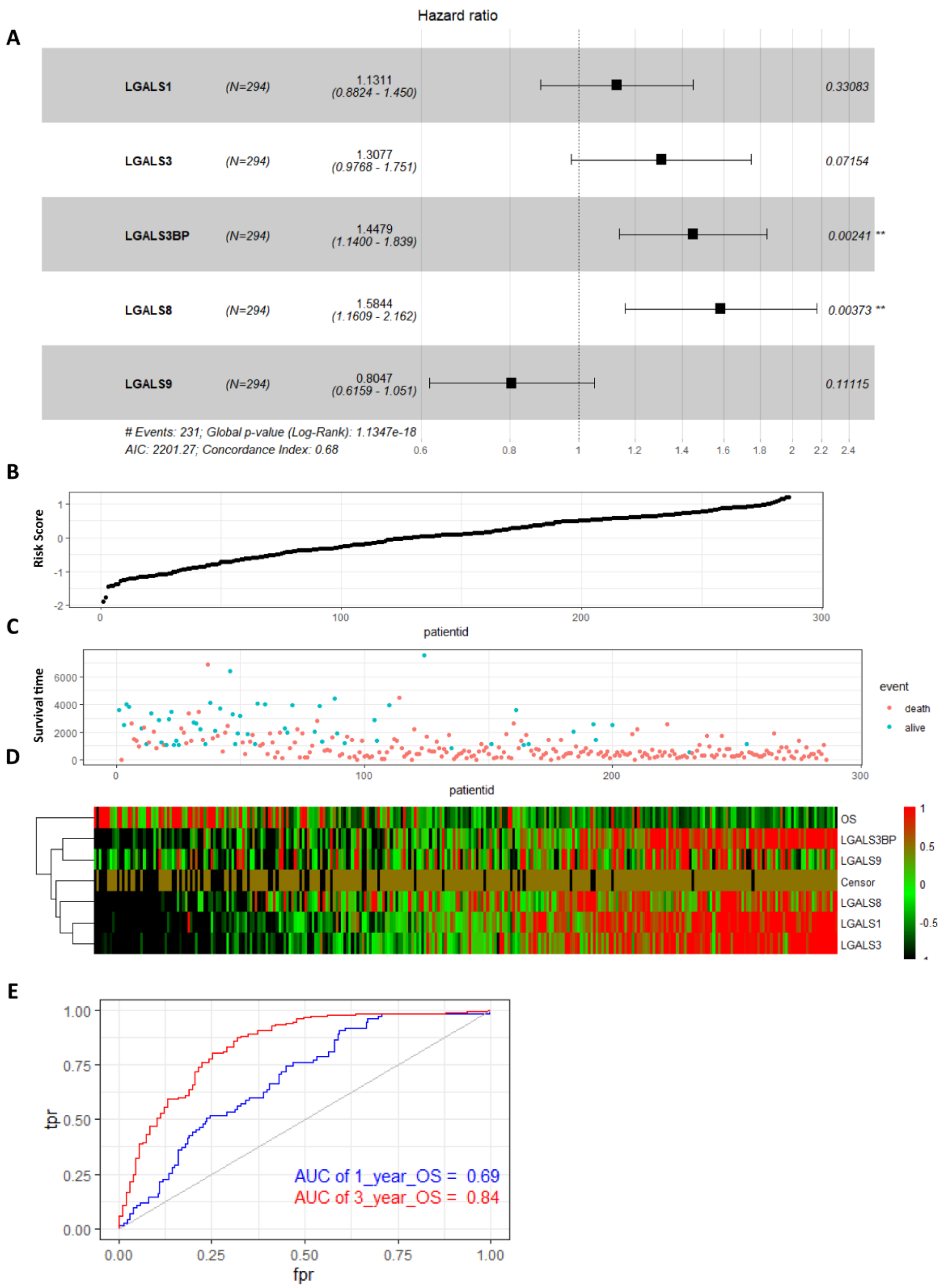

Supplementary Figure 5. Distribution of risk score, survival status, and risk heatmap of LGALS in patients with glioma revealed by multivariable Cox regression analysis of Rembrandt Glioma dataset. **(A)** Multivariable Cox regression analysis based on the 5 *LGALS*s expression. The black and solid squares represent the HR of death. Close-ended horizontal lines represent 95% CI. **(B)** The risk score curve of the *LGALS*s signature. **(C)** Patient survival status and time distributed by risk score. **(D)** Heatmaps of the expression levels of the 5 *LGALS*s and OS time of glioma patients. The colors from green to red indicate the expression level from low to high. **(E)** The prognostic performance of the *LGALS*s signature demonstrated by the time-dependent ROC curve for predicting the 1-, 3-year OS rates in the Rembrandt Glioma dataset.

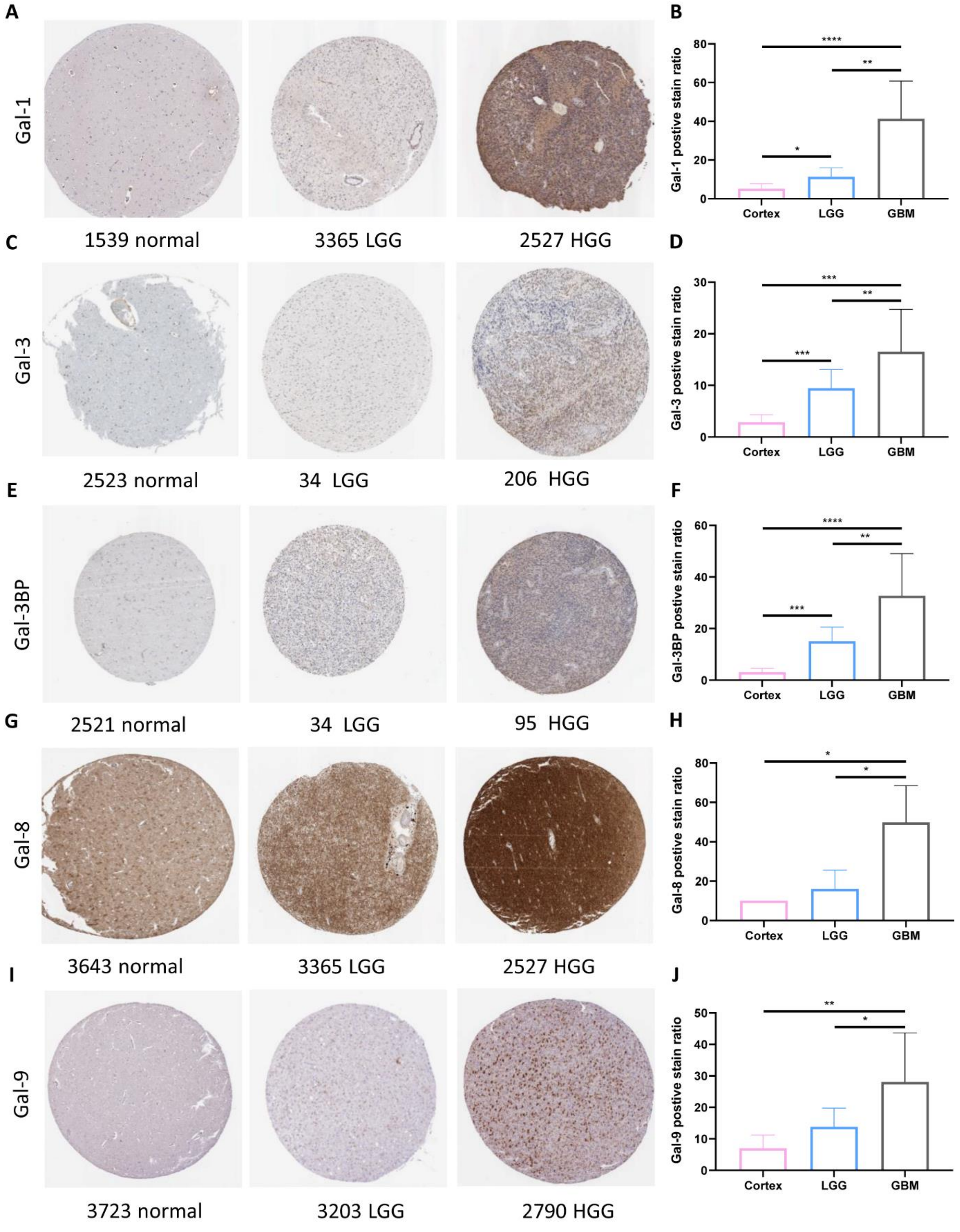

Supplementary Figure 6. Galectins expression in human normal brain, LGG and HGG tissue as shown by IHC staining of HPA database. (A) IHC staining of Gal-1 in glioma patients of HPA database. Representative staining images of normal brain, LGG and HGG are shown. **(B)** The positive stain ratio of Gal-1 in HPA glioma specimens, the statistical chart of Figure S6A. Similarly, the representative IHC staining images of Gal-3 **(C)** and statistical chart of its positive stain ratio **(D)**,

the representative IHC staining images of Gal-3BP **(E)** and statistical chart of its positive stain ratio **(F)**, the representative IHC staining images of Gal-8 **(G)** and statistical chart of its positive stain ratio **(H)**, the representative IHC staining images of Gal-9 **(I)** and statistical chart of its positive stain ratio **(J)** were shown.

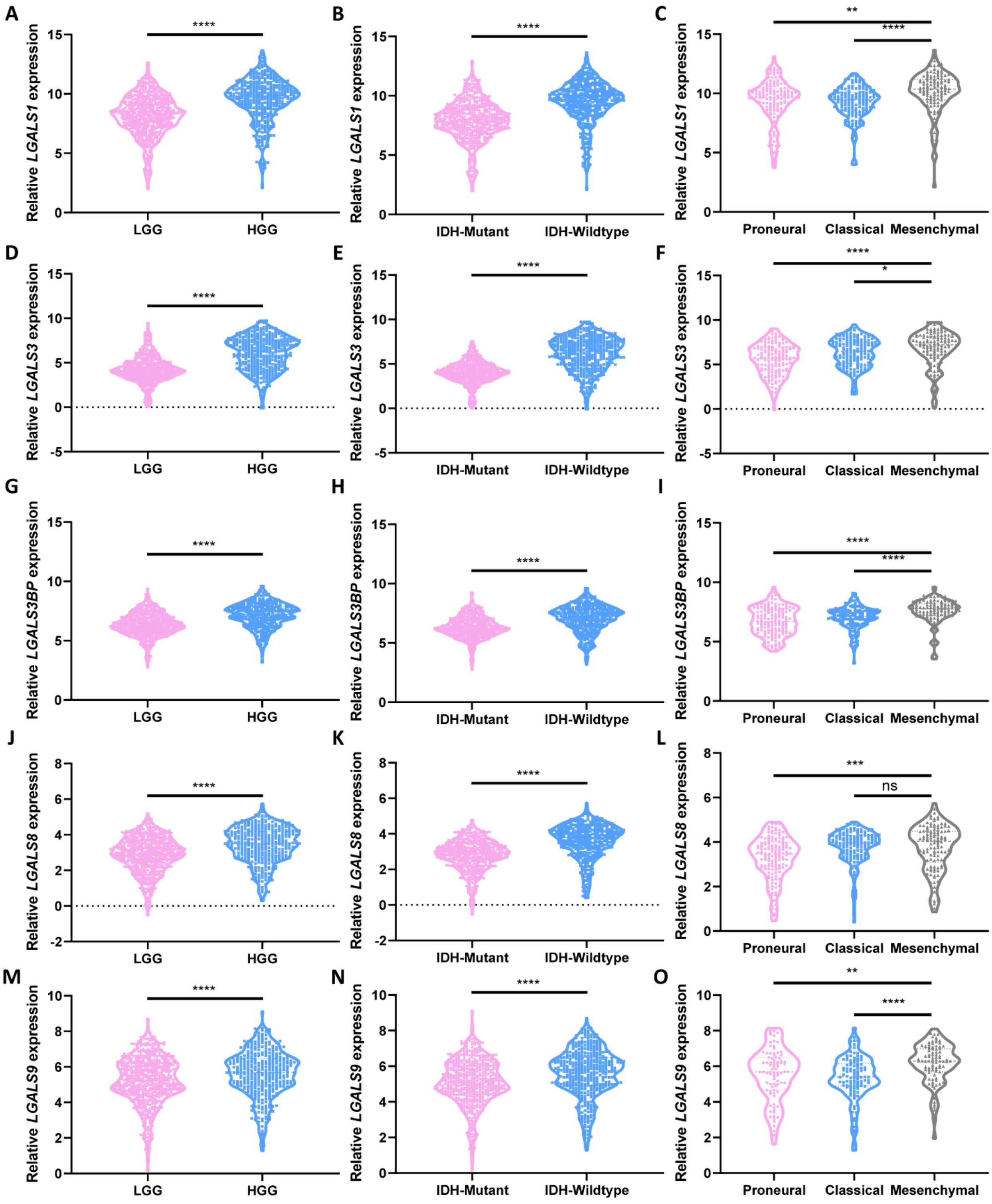

Supplementary Figure 7. LGALS expression correlates with glioma grades, GBM subtypes, and

IDH mutation status in CGGA Glioma dataset. **(A)** Relative *LGALS*1 expression level of LGG and HGG patients in CGGA Glioma dataset. **(B)** Relative *LGALS*1 expression level of IDH-Mutant and IDH-Wildtype patients in CGGA Glioma dataset. **(C)** Relative *LGALS*1 expression level of different molecular subtype patients in CGGA Glioma dataset. **(D-F)** Relative *LGALS*3 expression level of LGG and HGG patients **(D)**, IDH-Mutant and IDH-Wildtype patients **(E)**, different molecular subtype patients **(F)** in CGGA Glioma dataset. **(G-I)** Relative *LGALS*3BP expression level of LGG and HGG patients **(G)**, IDH-Mutant and IDH-Wildtype patients **(H)**, different molecular subtype patients **(I)** in CGGA Glioma dataset. **(J-L)** Relative *LGALS*8 expression level of LGG and HGG patients **(J)**, IDH-Mutant and IDH-Wildtype patients **(K)**, different molecular subtype patients **(L)** in CGGA Glioma dataset. **(M-O)** Relative *LGALS*3 expression level of LGG and HGG patients **(M)**, IDH-Mutant and IDH-Wildtype patients **(N)**, different molecular subtype patients **(O)** in CGGA Glioma dataset.

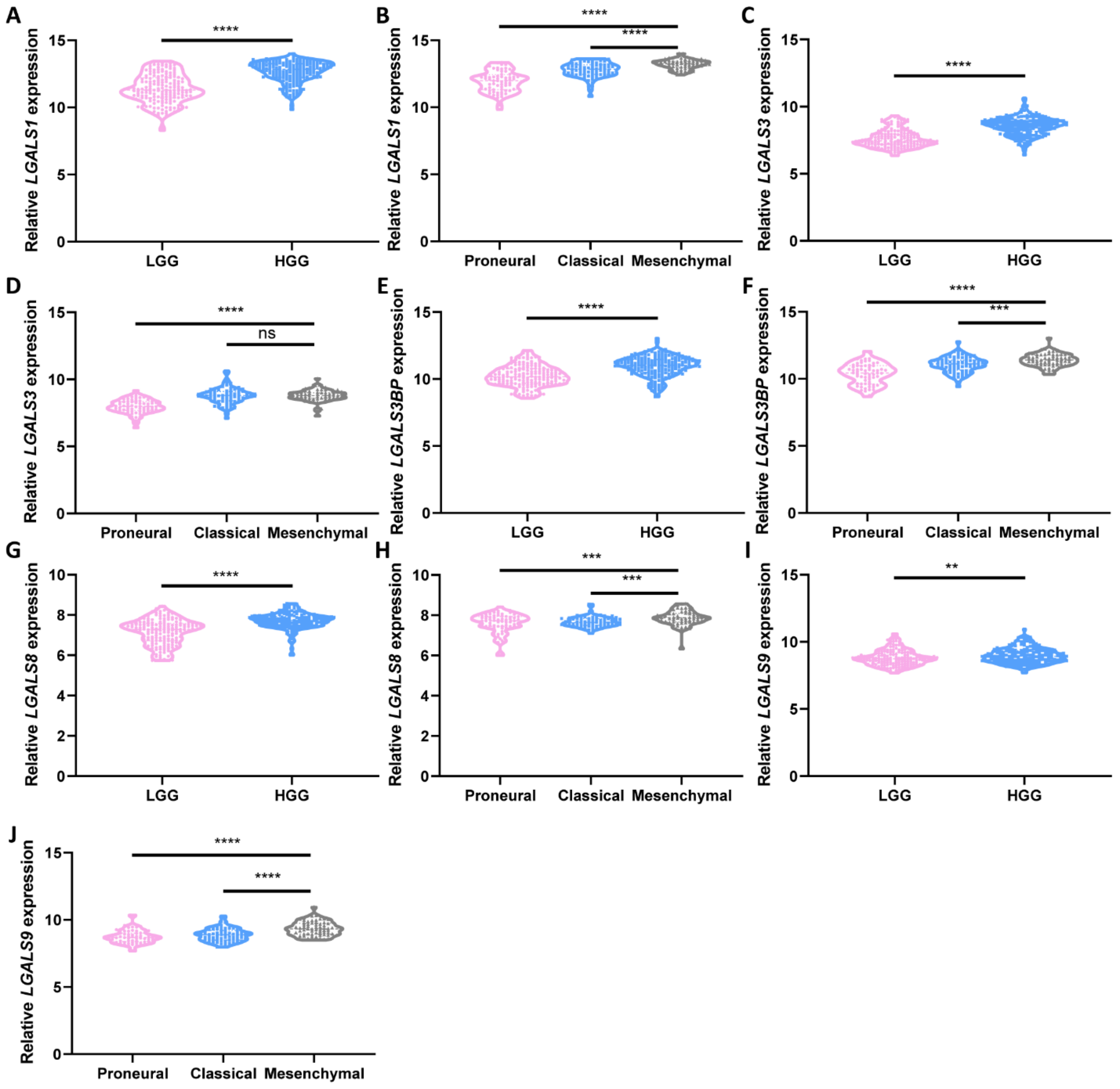

Supplementary Figure 8. LGALS expression correlates with glioma grades, GBM subtypes, and IDH mutation status in Rembrandt Glioma dataset. **(A)** Relative *LGALS*1 expression level of LGG and HGG patients in Rembrandt Glioma dataset. **(B)** Relative *LGALS*1 expression level of different molecular subtype patients in Rembrandt Glioma dataset. **(C-D)** Relative *LGALS*3 expression level of LGG and HGG patients **(C)**, different molecular subtype patients **(D)** in Rembrandt Glioma dataset. **(E-F)** Relative *LGALS*3BP expression level of LGG and HGG patients **(E)**, different molecular subtype patients **(F)** in Rembrandt Glioma dataset. **(G-H)** Relative *LGALS*8 expression level of LGG and HGG patients **(G)**, different molecular subtype patients **(H)** in Rembrandt Glioma dataset. **(I-J)** Relative *LGALS*3 expression level of LGG and HGG patients **(I)**, different molecular subtype patients **(J)** in Rembrandt Glioma dataset.

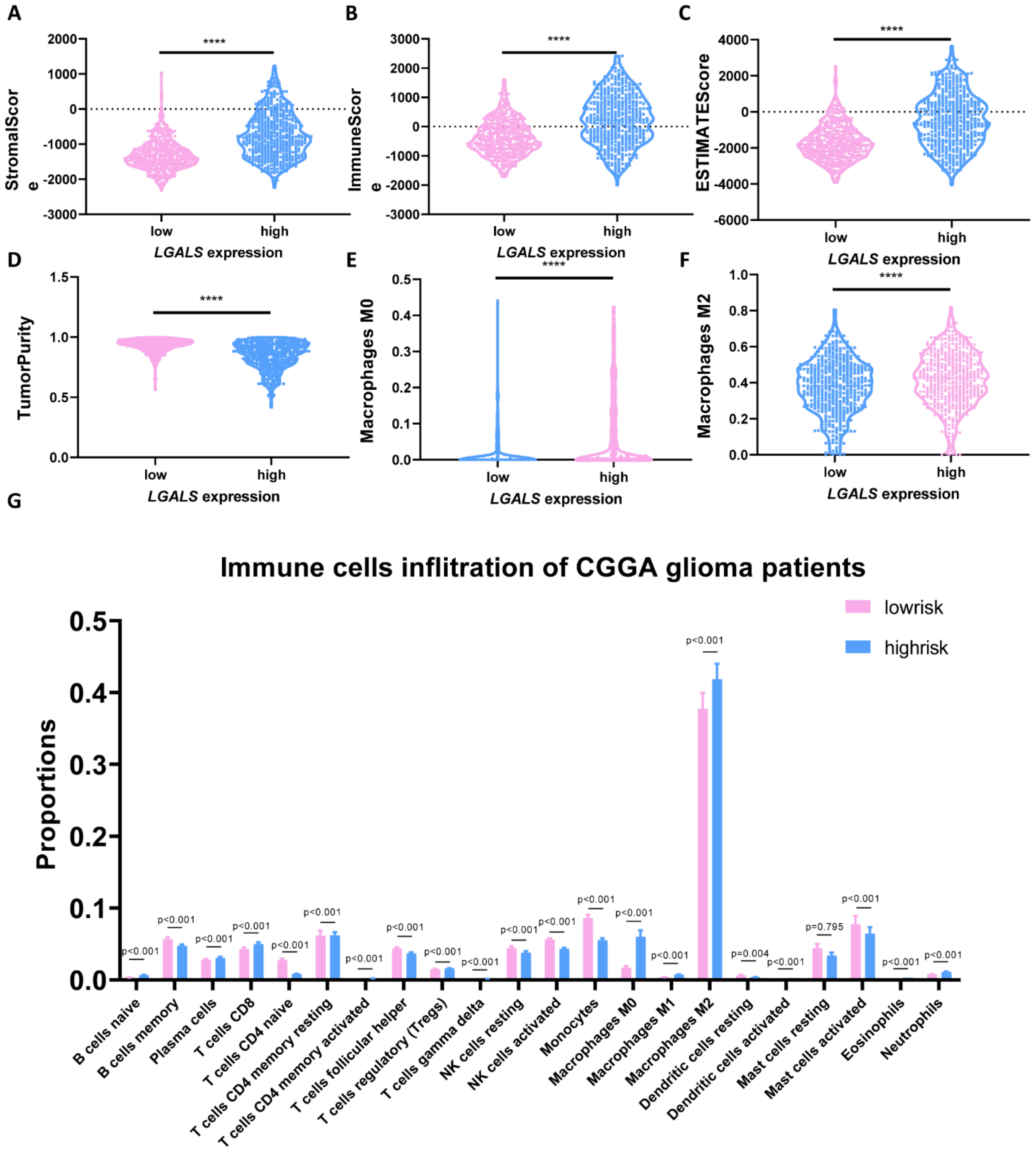

Supplementary Figure 9. LGALS expression indicates stromal cell infiltration and immunosuppression in CGGA Glioma dataset. **(A)** The StromalScore, **(B)** ImmuneScore, **(C)** ESTIMATEScore and **(D)** TumorPurity of glioma patients with high and low *LGALS*s expression based on *LGALS*s signature. **(G)** The immune cells infiltration of glioma patients with high and low *LGALS*s expression based on *LGALS*s signature, the proportions of 22 immune cells were calculated. The proportion of Macrophage M2 **(E)** and CD8^{+} T cell **(F)** was shown separately.

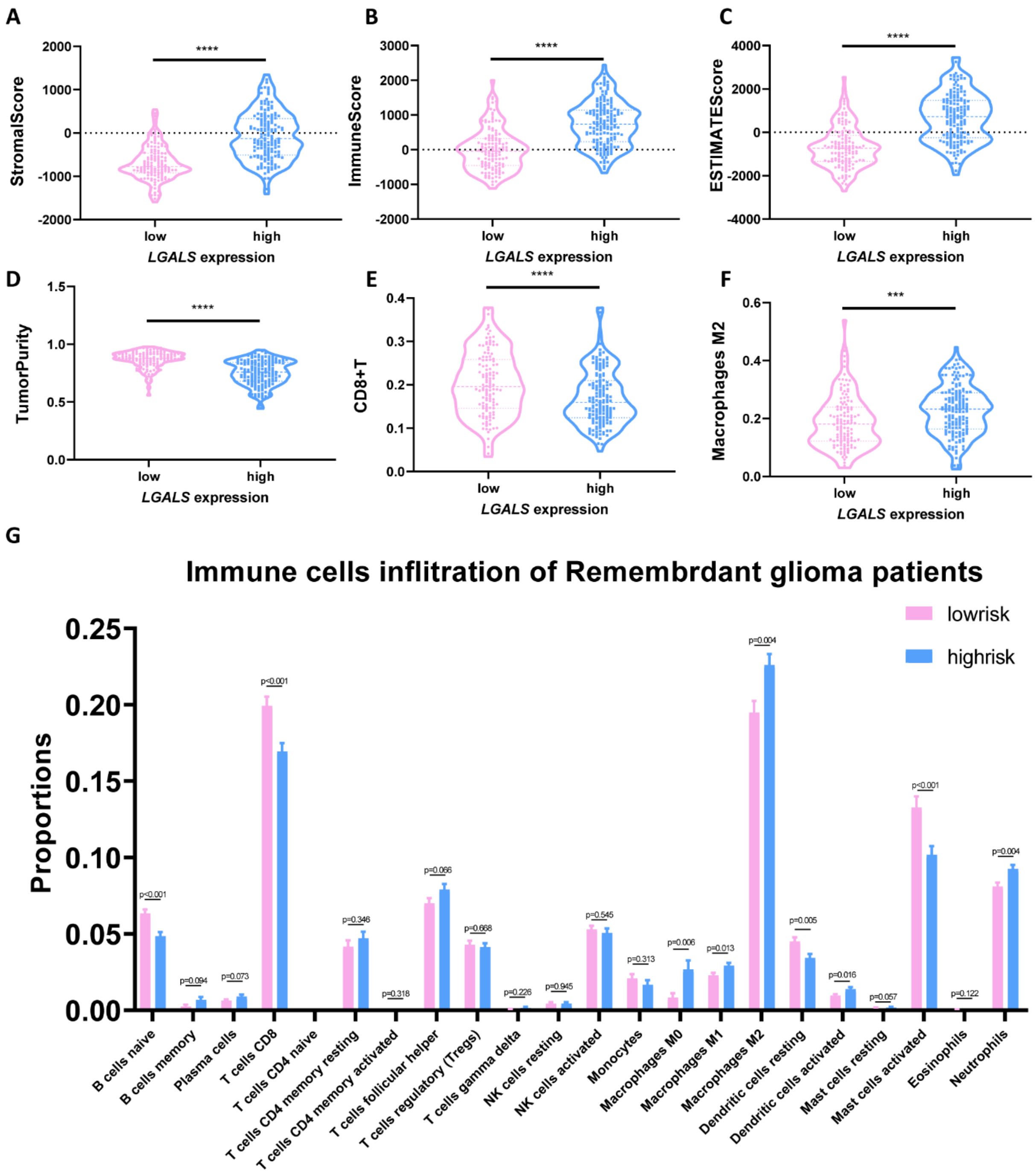

Supplementary Figure 10. LGALS expression indicates stromal cell infiltration and immunosuppression in Rembrandt Glioma dataset. **(A)** The StromalScore, **(B)** ImmuneScore, **(C)** ESTIMATEScore and **(D)** TumorPurity of glioma patients with high and low *LGALS*s expression based on *LGALS*s signature. **(G)** The immune cells infiltration of glioma patients with high and low *LGALS*s expression based on *LGALS*s signature, the proportions of 22 immune cells were calculated. The proportion of Macrophage M2 **(E)** and CD8^{+} T cell **(F)** was shown separately.

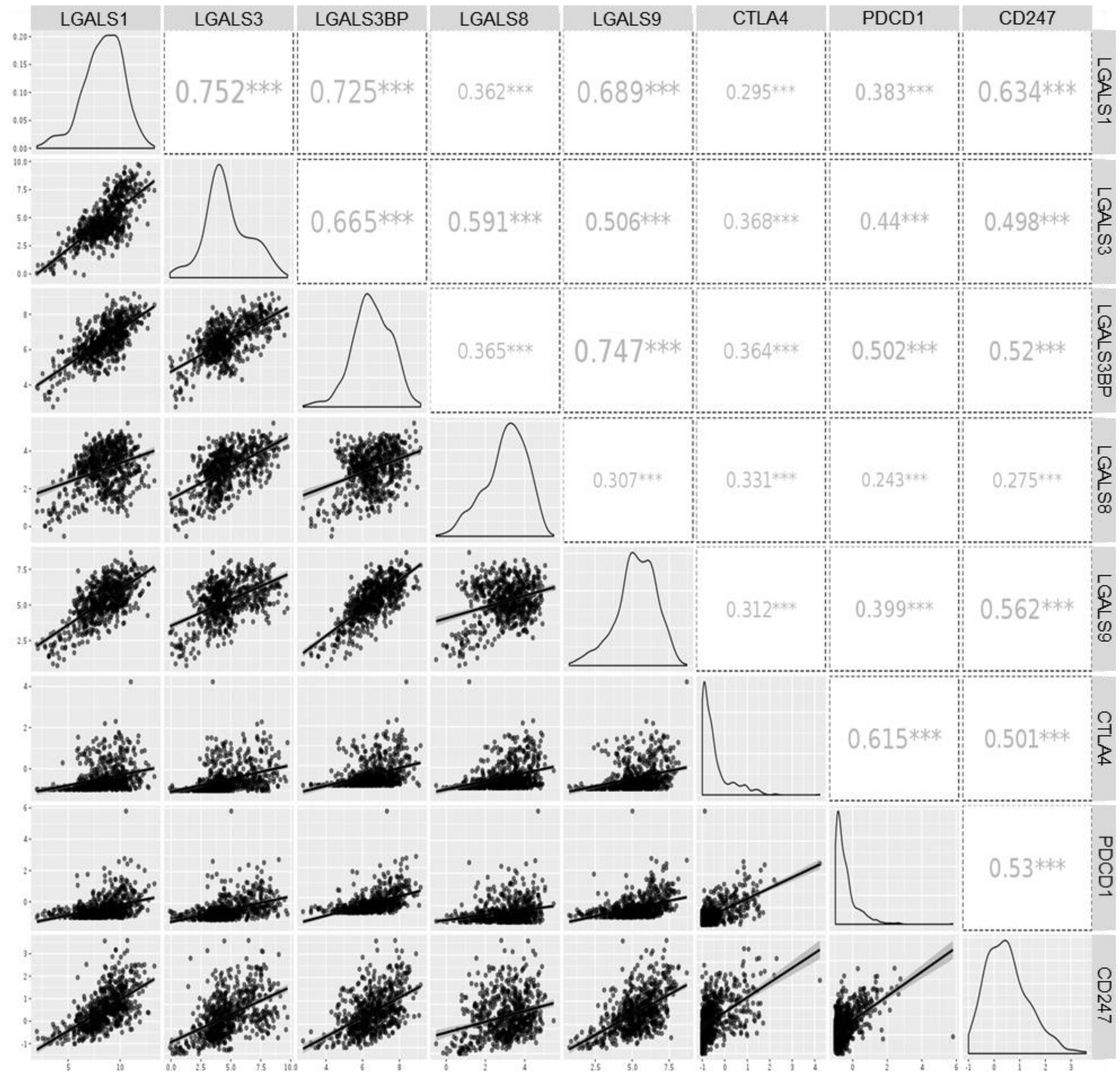

Supplementary Figure 11. Correlations between LGALSs and immune checkpoint markers expression in CGGA Glioma dataset

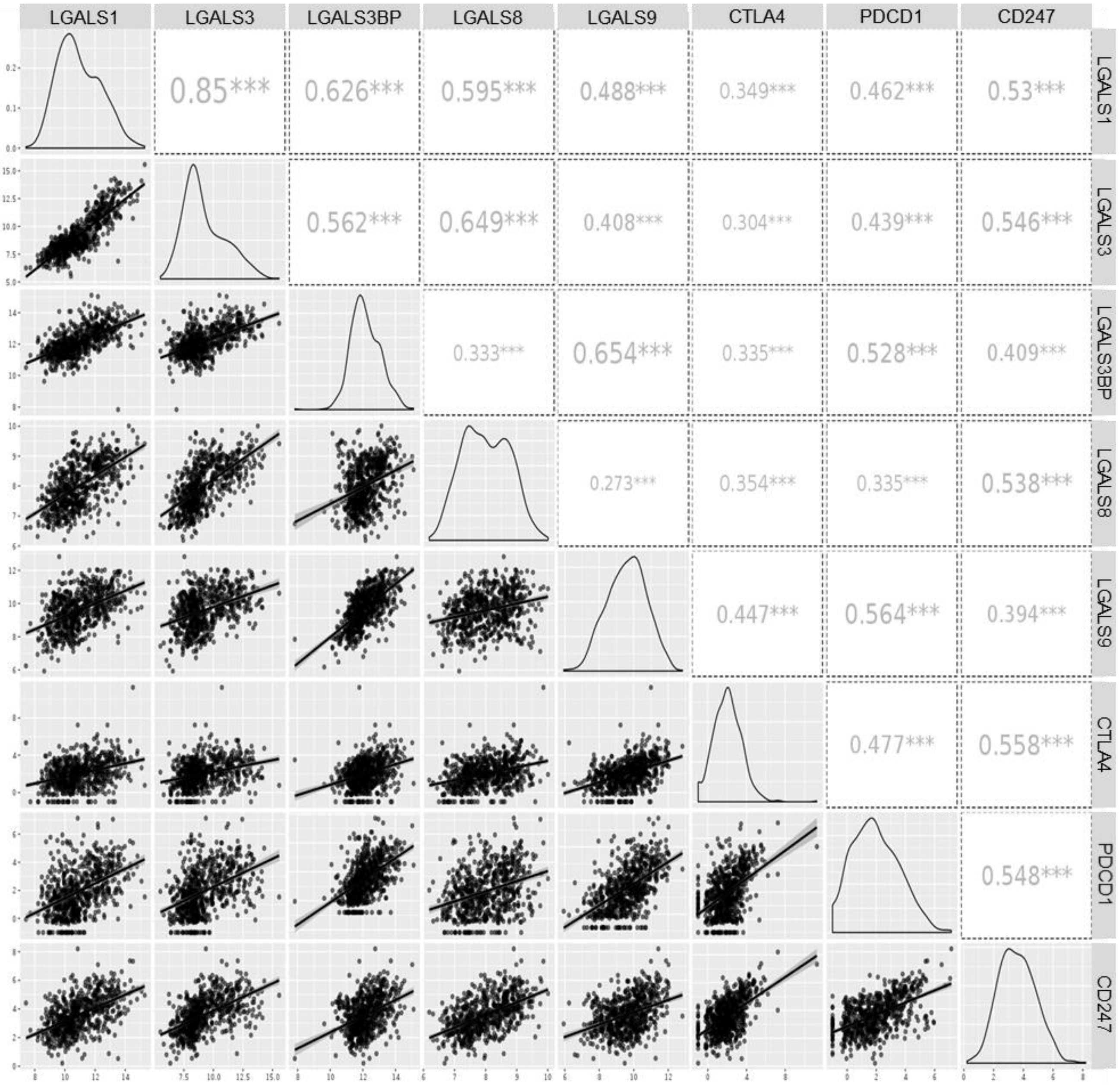

Supplementary Figure 12. Correlations between LGALSs and immune checkpoint markers expression in TCGA Glioma dataset

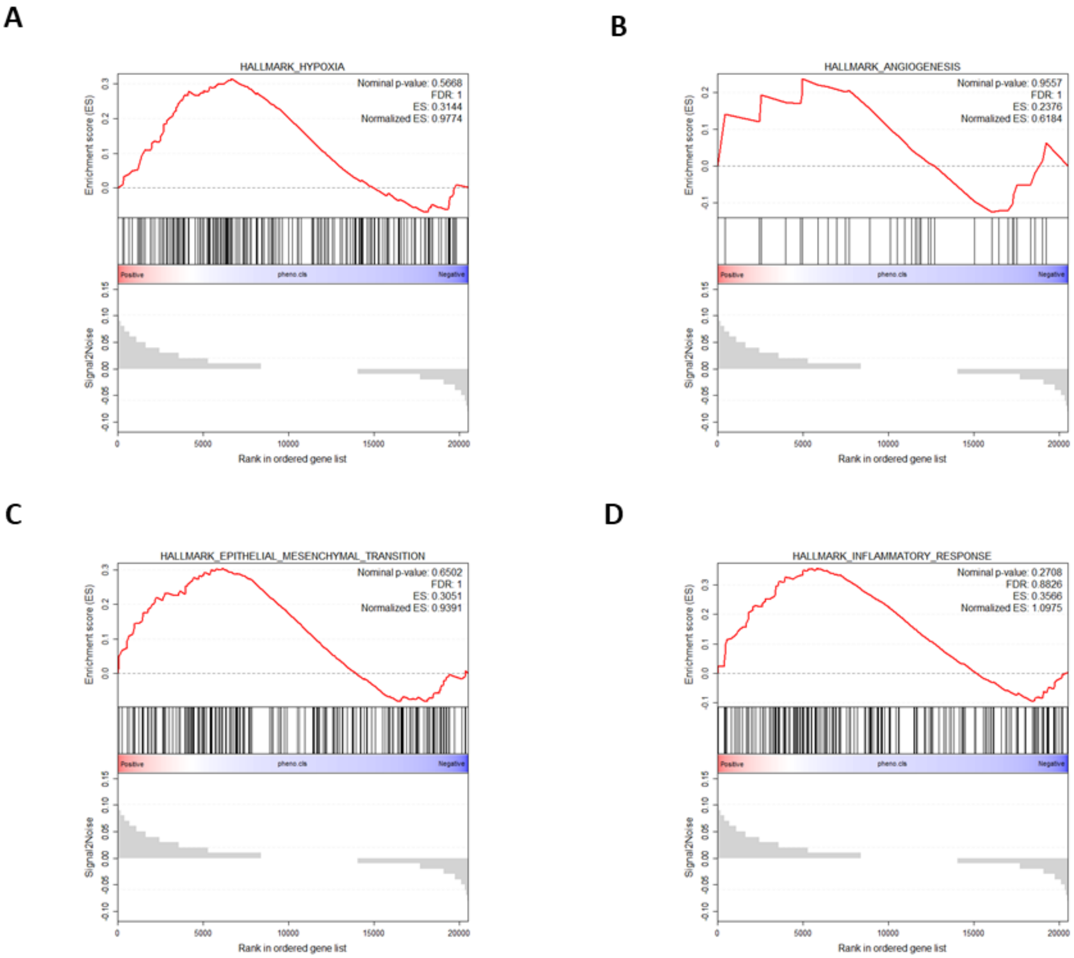

Supplementary Figure 13. GSEA analysis of LGALSs expression in TCGA dataset. GSEA analysis of TCGA dataset shows that pathways containing hypoxia **(A)**, angiogenesis **(B)**, epithelial to mesenchymal transition (EMT) **(C)**, and inflammatory response**(D)** were activated in patients with high risk-scores

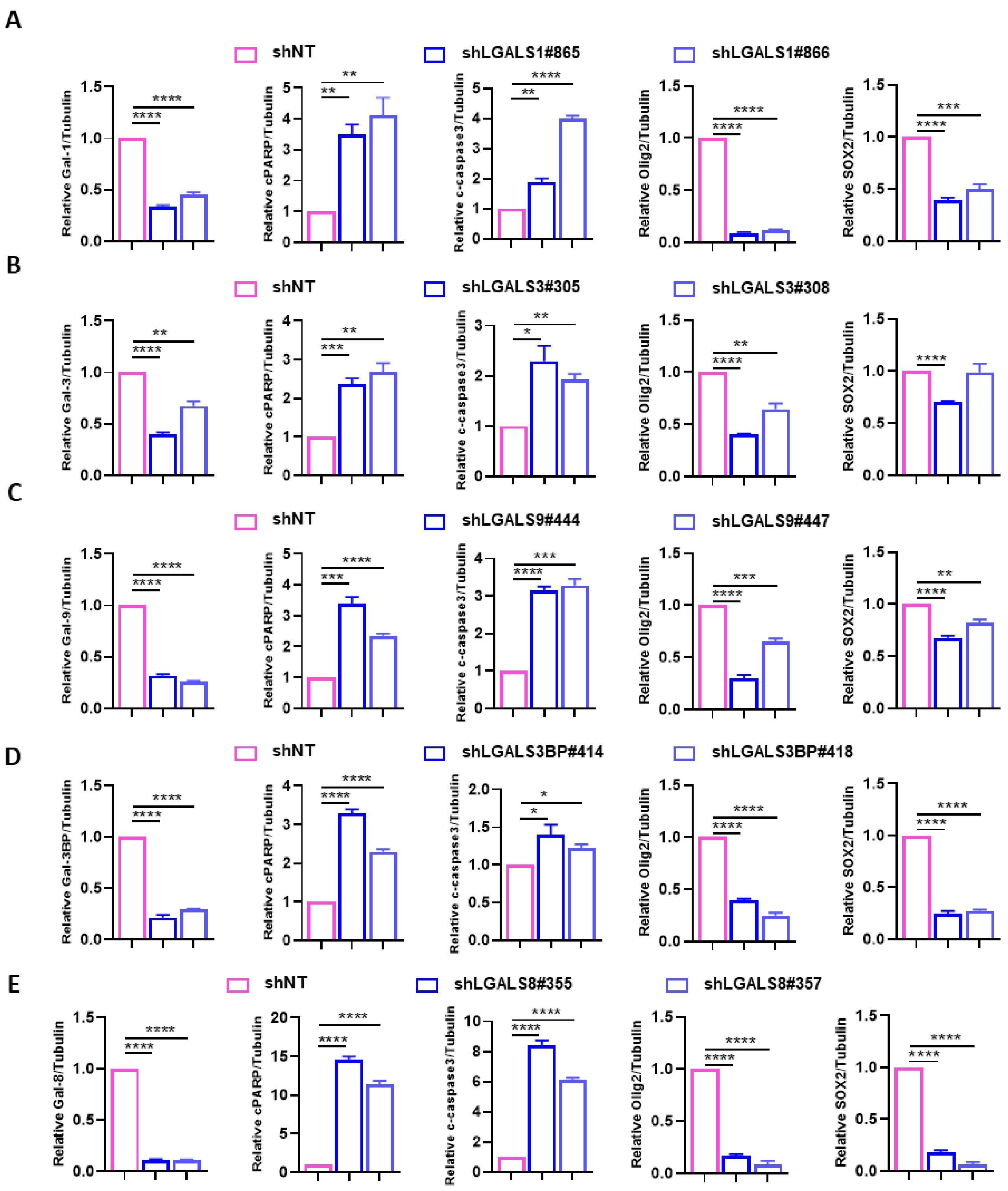

Supplementary Figure 14. Galectins Promote T387-GSC stemness maintenance and proliferation in vitro. **(A-E)** Grayscale analyses and statistical analyses of bands in Figure 8U-Y.

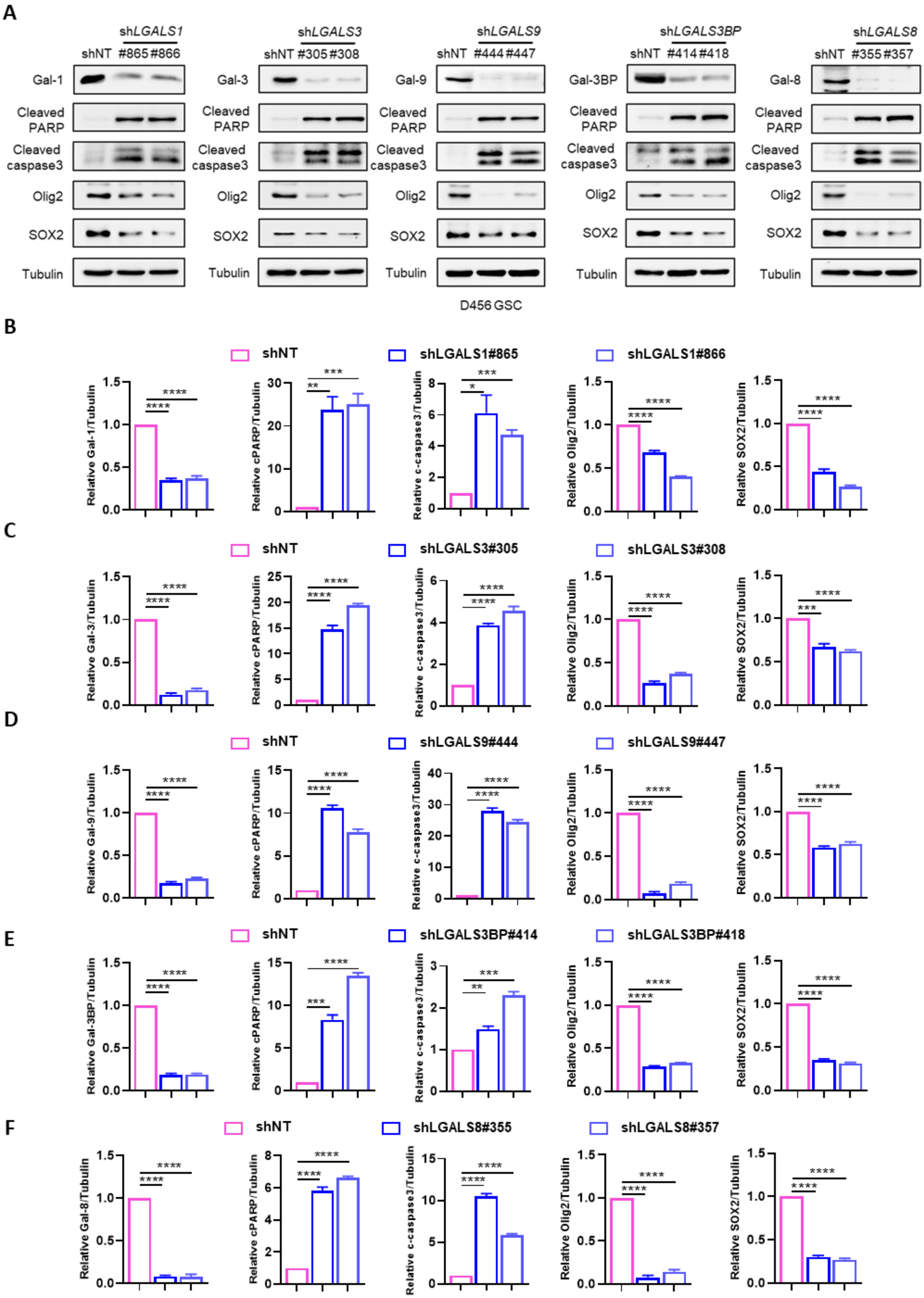
A
shLGALS1
shNT #865 #866
Gal-1
Cleaved PARP
Cleaved caspase3
Olig2
SOX2
Tubulin
shLGALS3
shNT #305 #308
Gal-3
shLGALS9
shNT #444 #447
Gal-9
shLGALS3BP
shNT #414 #418
Gal-3BP
shLGALS8
shNT #355 #357
Gal-8
D456 GSC
B
shNT
shLGALS1#865
shLGALS1#866
Relative Gal-1/Tubulin
Relative cPARP/Tubulin
Relative c-caspase3/Tubulin
Relative Olig2/Tubulin
Relative SOX2/Tubulin
C
shNT
shLGALS3#305
shLGALS3#308
Relative Gal-3/Tubulin
D
shNT
shLGALS9#444
shLGALS9#447
Relative Gal-9/Tubulin
E
shNT
shLGALS3BP#414
shLGALS3BP#418
Relative Gal-3BP/Tubulin
F
shNT
shLGALS8#355
shLGALS8#357
Relative Gal-8/Tubulin

Supplementary Figure 15. Galectins Promote D456-GSC stemness maintenance and proliferation in vitro. **(A)** Western blotting analysis of cleaved PARP, cleaved caspase3, Olig2 and SOX2 proteins in D456 GSC with LGALS1, LGALS3, LGALS3BP, LGALS8, LGALS9 knockdown. **(B-F)** Grayscale analyses and statistical analyses of bands in **(A)**.
